# Supplementary figures and images for: Genome-wide analysis of DNA replication and DNA double-strand breaks using TrAEL-seq
Source: PLoS Biol. 2021 Mar 24;19(3):e3000886. doi: 10.1371/journal.pbio.3000886 (PMC8021198; doi:10.1371/journal.pbio.3000886)

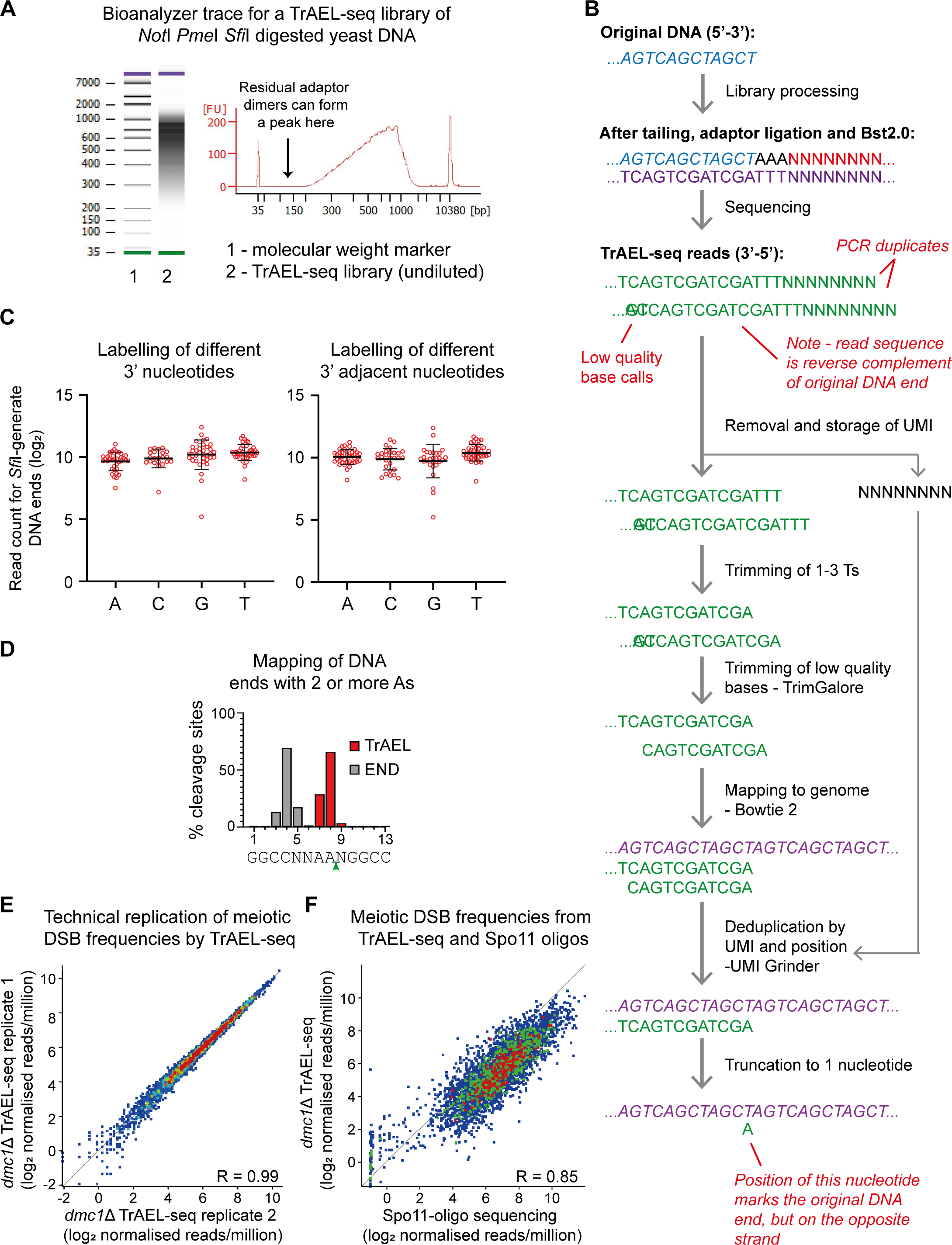

Supplement: S1 Fig — (A) Example Bioanalyzer trace for the amplified library of NotI PmeI SfiI-digested yeast genomic DNA. A volume of 1 μl of the 10.5 μl final library was run on a DNA high sensitivity Bioanalyzer chip. This shows a complete absence of adaptor or primer dimers, which is only achieved after 2 successive AMPure purifications. This trace is typical for TrAEL-seq libraries. (B) Schematic of TrAEL-seq read processing pathway. TrAEL-seq reads are the reverse complement of the original DNA end. The 8 nucleotide UMI is removed and stored, then up to 3 T’s are removed from the 5′ of the read. Poor-quality reads and adaptor sequences are removed by TrimGalore, then reads are mapped using Bowtie 2. Deduplication is performed based on the UMI and the mapped start site by UMI grinder, then the reads are finally truncated to a single nucleotide representing the reverse complement of the terminal nucleotide of the original DNA strand. (C) Quantitation of DNA ends generated by SfiI digestion categorised by the 3′ nucleotide or the nucleotide adjacent to the 3′ nucleotide in TrAEL-seq data. Bars show mean and 1 SD. (D) Precision mapping of SfiI cleavage sites by TrAEL-seq and END-seq, as Fig 1D. This graph represents the 10 SfiI sites that have 2 or more As at the 3′ end (GGCCNNAA|NGGCC). In this category are 5 ends with 2 As, 2 ends with 3 As, and 3 ends with 4 As. Mapped locations of 3′ ends were averaged across each category of site and expressed as a percentage of all 3′ ends mapped by each method to that category of site. (E) Scatter plot of log-transformed normalised read counts at all 3,907 Spo11 cleavage hotspots annotated by Mohibullah and Keeney [1], comparing 2 technical replicate TrAEL-seq libraries generated from the same sample of dmc1Δ cells. The 2 libraries were prepared approximately 6 months apart by 2 different researchers from cells stored in 70% ethanol at −70°. (F) Scatter plot of log-transformed normalised read counts at all 3,907 Spo11 cleavage hotspots annotate [file pbio.3000886.s001.tif]

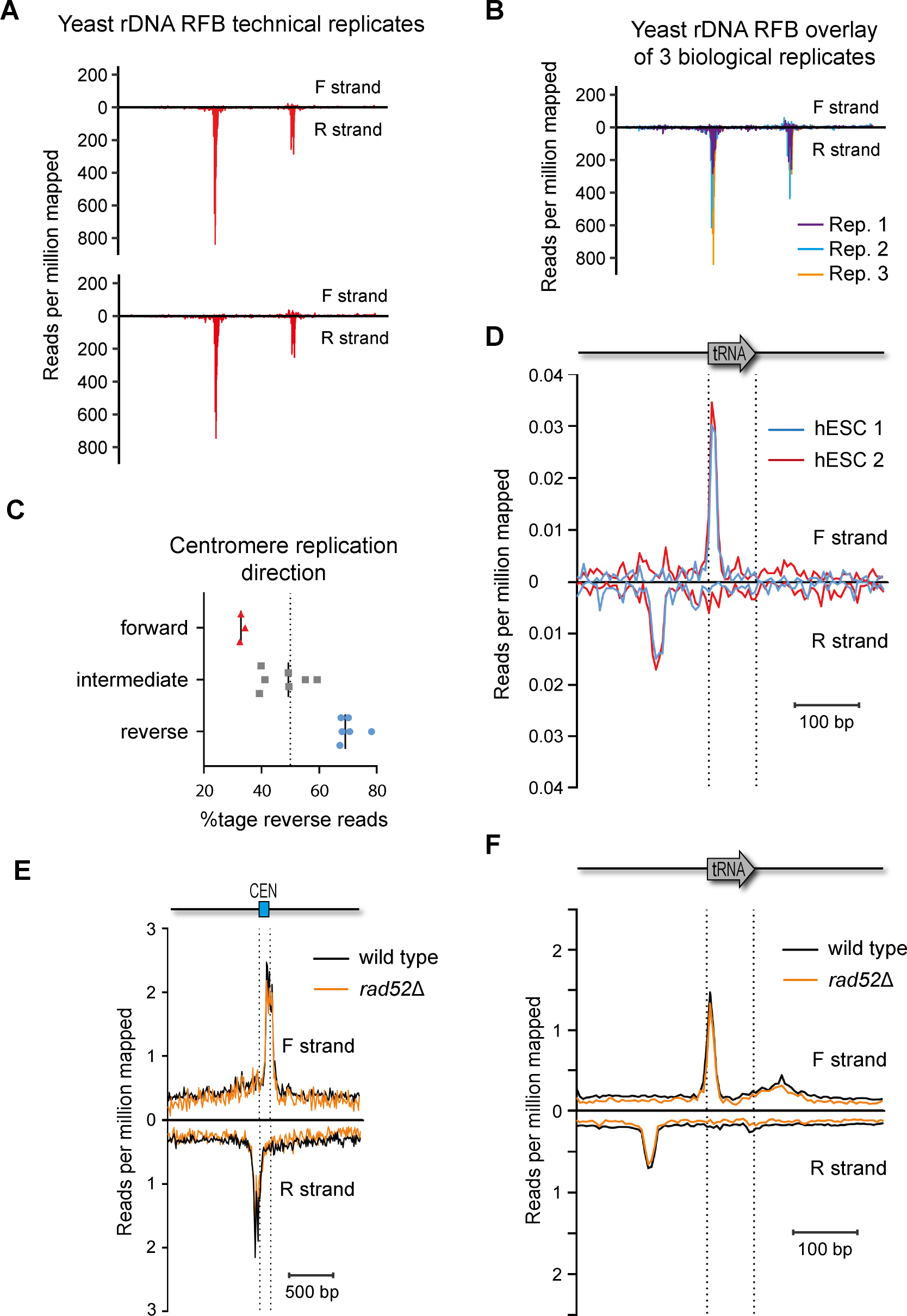

Supplement: S2 Fig — (A) Reproducibility of RFB detection between 2 technical replicates. The 2 libraries were prepared approximately 6 months apart by 2 different researchers from cells stored in 70% ethanol at −70°. (B) Detection of RFB peaks without nonreproducible background peaks in 3 biological replicates TrAEL-seq libraries derived from wild-type cells. (C) Replication direction of centromeres, calculated based on the cdc9-AID GLOE-seq data (SRA accession: SRX6436838). Percentage of reverse reads was determined in the regions −1000 to −500 bp and +500 to +1000 bp relative to the annotated centromere, and the average of these values plotted. The region from −500 to +500 bp was excluded as replication fork stalling in this region obscures the replication direction. CEN2 is misleading as it is directly adjacent to a replication origin—see S1 File for profiles of individual centromeres. (D) Average TrAEL-seq profiles across tRNAs ±200 bp for 2 biological replicates of hESC cells, each averaged from 2 technical replicates. Reads are separated by orientation on forward or reverse strands; all tRNAs are included. Read counts per million reads mapped were calculated in nonoverlapping 5 bp bins. (E) Average TrAEL-seq profiles across all centromeres ±1 kb for wild-type and rad52Δ cells. Read counts per million reads mapped were calculated in nonoverlapping 10 bp bins. (F) Average TrAEL-seq profiles across all tRNAs ±200 bp for wild-type and rad52Δ cells. Read counts per million reads mapped were calculated in nonoverlapping 5 bp bins. Numerical data underlying this figure can be found in S2 Data. hESC, human embryonic stem cell; RFB, replication fork barrier; TrAEL-seq, Transferase-Activated End Ligation sequencing. (TIF) [file pbio.3000886.s002.tif]

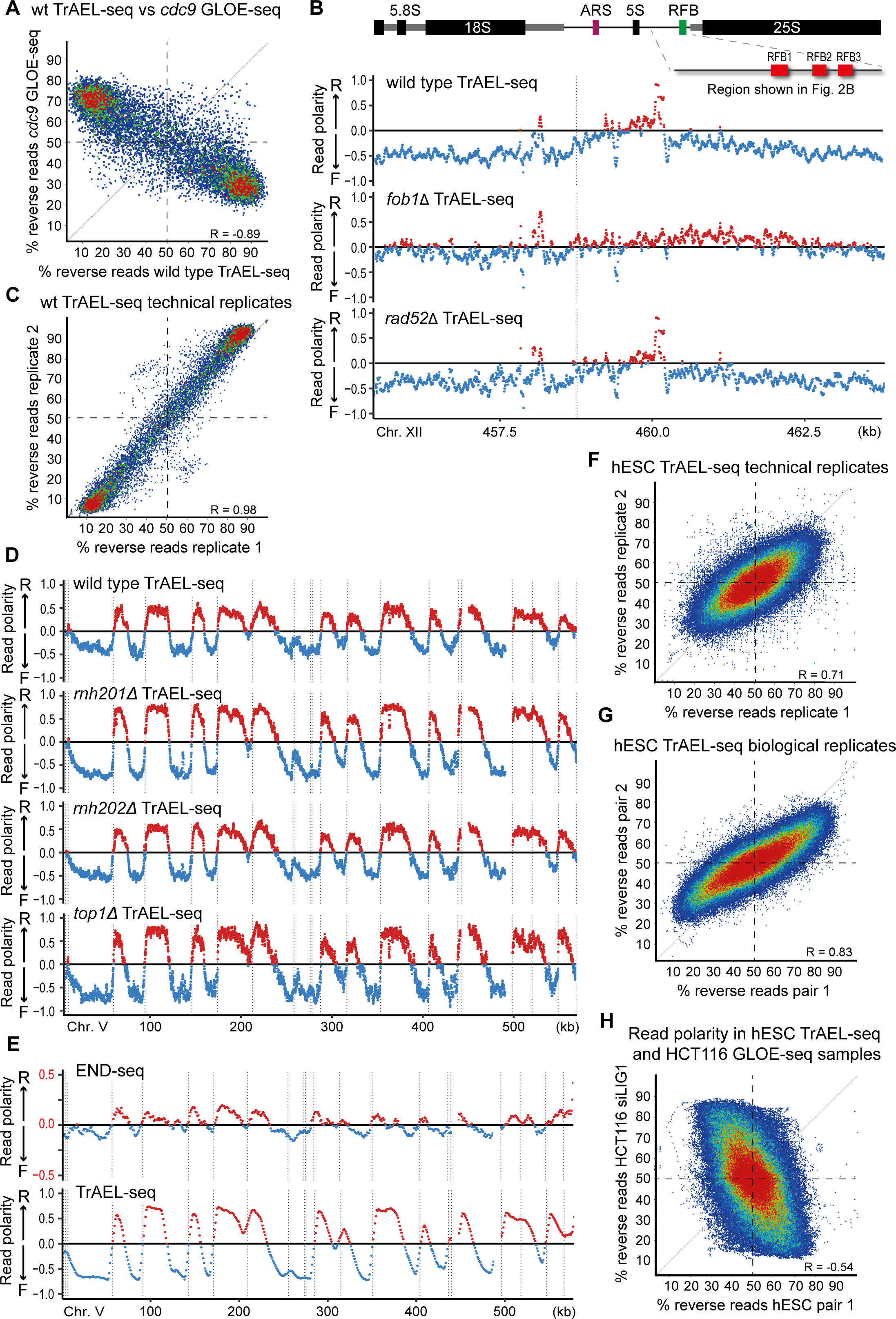

Supplement: S3 Fig — (A) Scatter plot showing the percentage of reverse reads compared to all reads in 1 kb genomic windows spaced every 1 kb, comparing TrAEL-seq data from wild-type cells and GLOE-seq data from Cdc9-depleted cells (SRA accession: SRX6436838). (B) Read polarity plots showing TrAEL-seq data for wild type, fob1Δ, and rad52Δ across a single rDNA repeat. The 35S rRNA gene transcribed by RNA polymerase I is shown as a thicker grey line and is transcribed right to left in this representation. Mature rRNA genes are shown in black; the RFB and the ARS are also annotated. Inset is the region containing the RFB sites that is shown in Fig 2B. (C) Scatter plot showing the percentage of reverse reads compared to all reads in 1 kb genomic windows spaced every 1 kb, comparing TrAEL-seq data from 2 technical replicates of wild-type cells. (D) Read polarity plot across chromosome V for TrAEL-seq datasets of wild type compared to the RNase H2 mutants rnh201Δ and rnh202Δ and topoisomerase I mutant top1Δ. (E) Read polarity plot for chromosome V comparing END-seq and TrAEL-seq data generated from two-halves of an agarose plug containing 10 million wild-type 3xCUP1 cells grown in synthetic complete glucose media. Note that the scale for the END-seq data is expanded as the bias in read polarity is much smaller in END-seq libraries. (F) Scatter plot showing the percentage of reverse reads compared to all reads in 250 kb genomic windows spaced every 10 kb, comparing TrAEL-seq data for 2 technical replicates generated from the same hESC sample. (G) Scatter plot showing the percentage of reverse reads compared to all reads in 250 kb genomic windows spaced every 10 kb, comparing TrAEL-seq data for 2 biological replicates of hESCs, each averaged from 2 technical replicates. (H) Scatter plot showing the percentage of reverse reads compared to all reads in 250 kb genomic windows spaced every 10 kb, comparing TrAEL-seq data from hESC cells (average of 2 technical replicates) to GLOE-seq data from LIG1 [file pbio.3000886.s003.tif]

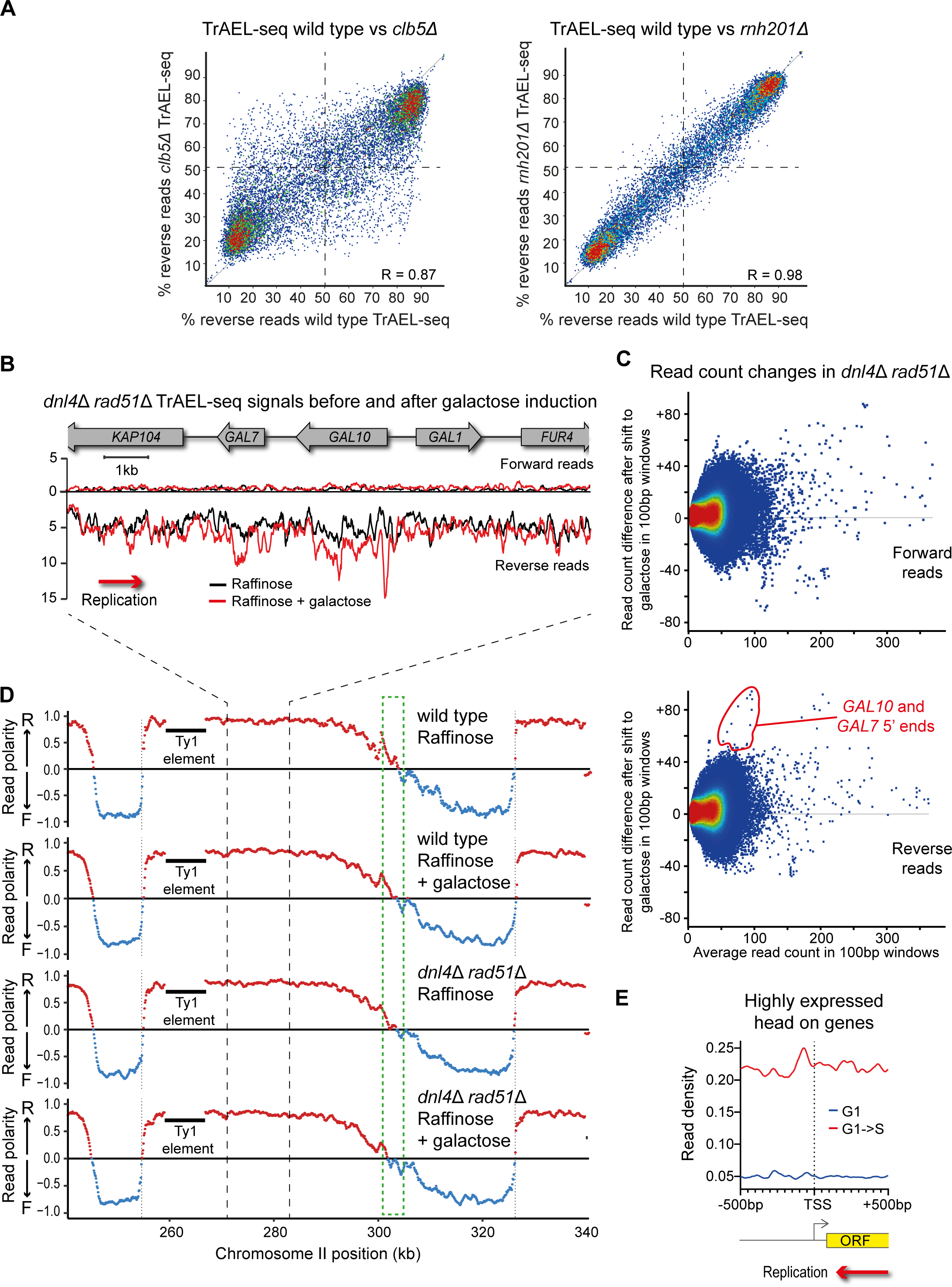

Supplement: S4 Fig — (A) Scatter plot showing the percentage of reverse reads compared to all reads in 1 kb genomic windows spaced every 1 kb, comparing TrAEL-seq data wild type and clb5Δ (left). An equivalent comparison between wild type and rnh201Δ (which has a wild-type replication profile) is shown for comparison (right). (B) Plot of read count across the GAL locus on galactose induction for dnl4Δ rad51Δ mutant, as Fig 4B. (C) MA plots of changing read count across the genome on galactose induction for dnl4Δ rad51Δ mutant, as Fig 4C. (D) Read polarity plots showing the replication profile of the region surrounding the GAL locus with and without galactose induction. Green box shows the site at which the replication fork which passes through the GAL locus encounters the oncoming fork from ARS211. (E) Plot of average TrAEL-seq read density around the TSS in the highest 25% expressed genes orientated head-on with replication (as Fig 4D). Data are shown for G1 and G1->S samples (Fig 3E); genes are averaged together within each sample, but the difference in average read count between samples is maintained. The nonreplicating G1 sample contains far less reads on average across TSS regions, and the peak upstream of the TSS is absent. Numerical data underlying this figure can be found in S7 Data. TrAEL-seq, Transferase-Activated End Ligation sequencing; TSS, transcriptional start site. (TIF) [file pbio.3000886.s004.tif]

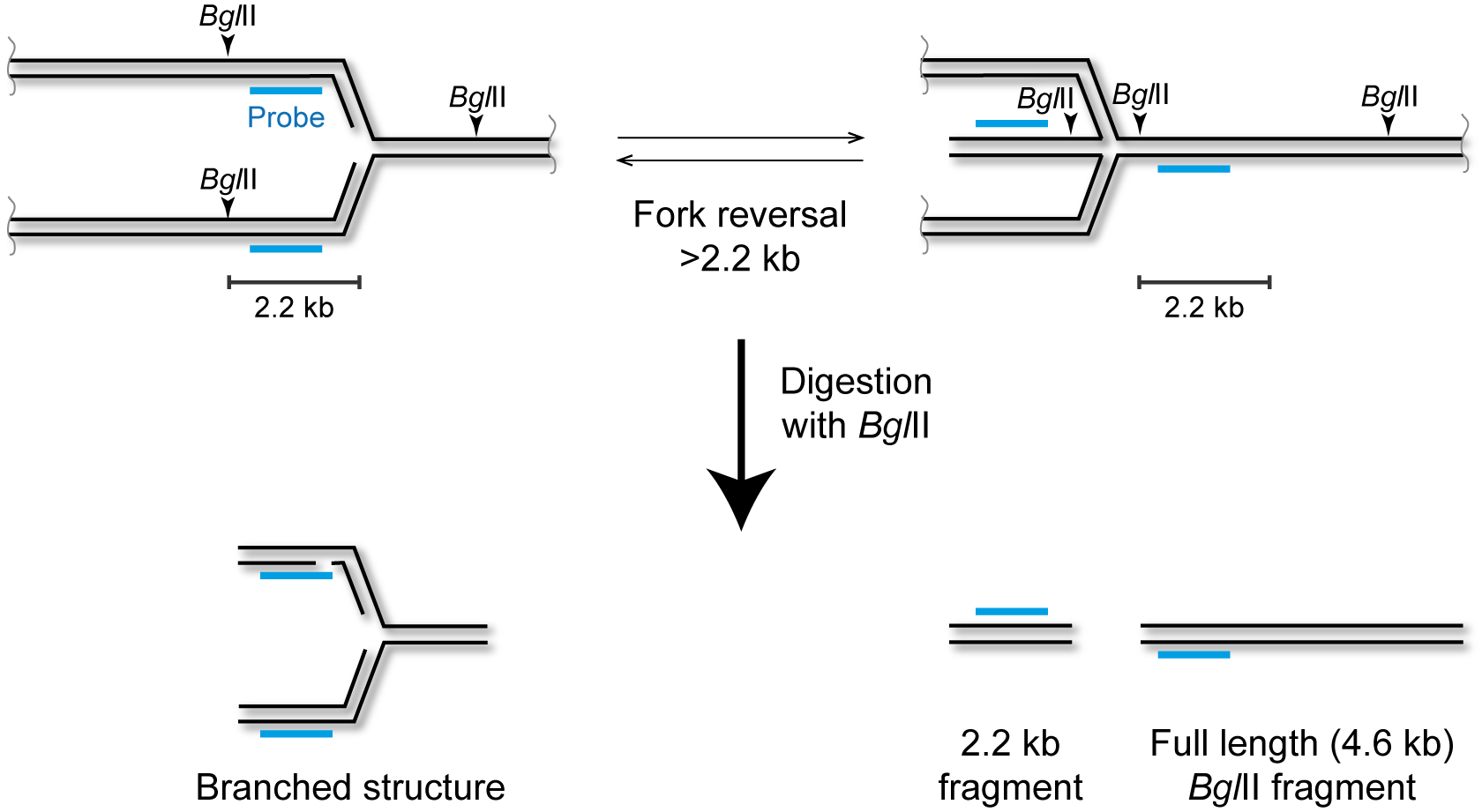

Supplement: S5 Fig — All Southern blot analyses that have reported direct detection of DSBs at RFBs utilise a restriction digestion to separate the region of interest. For the yeast RFB, to our knowledge, the enzyme used has always been BglII, the cleavage sites for which lie 2.2 kb and 2.4 kb each side of the RFB. Forks that reverse past the BglII site would yield a BglII fragment the same size (2.2 kb) as a fork that is cleaved at the RFB. Only fragments that would hybridise to the probe (blue) are shown. DSB, double-strand break; RFB, replication fork barrier. (TIF) [file pbio.3000886.s005.tif]
