# Supplementary material for: Genome-wide analysis of DNA replication and DNA double-strand breaks using TrAEL-seq
Source: PLoS Biol. 2021 Mar 24;19(3):e3000886. doi: 10.1371/journal.pbio.3000886 (PMC8021198; doi:10.1371/journal.pbio.3000886)

Centromeres replicated left to right

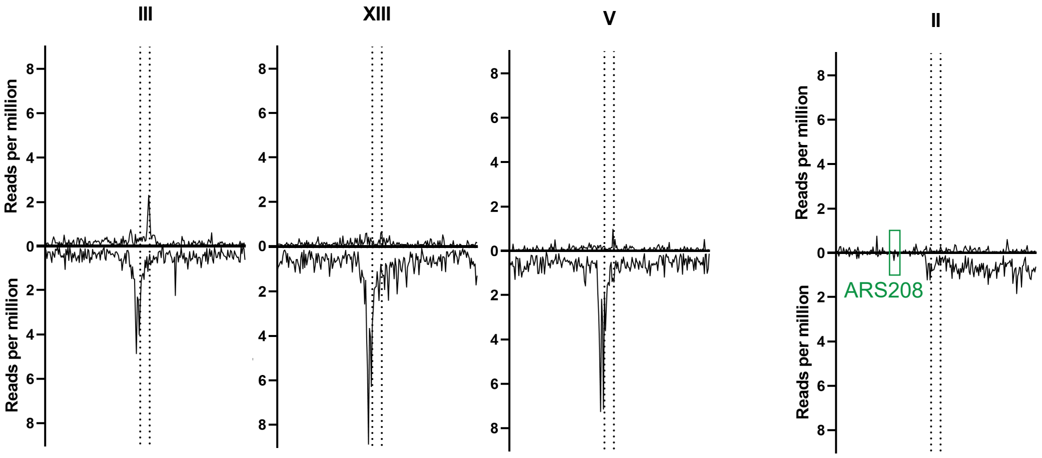

Centromeres replicated right to left

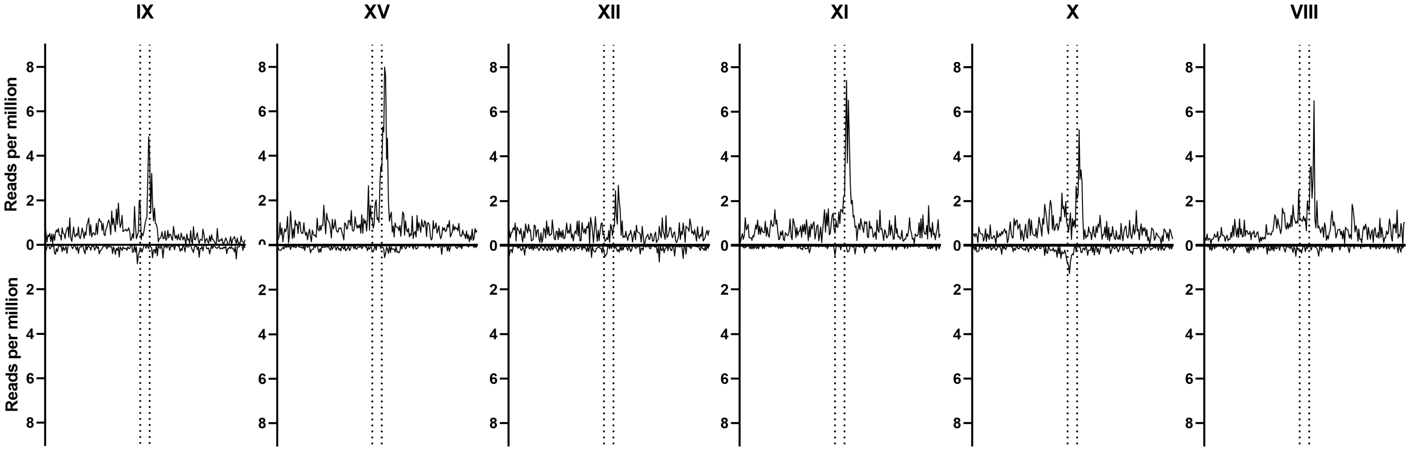

Centromeres replicated in both directions

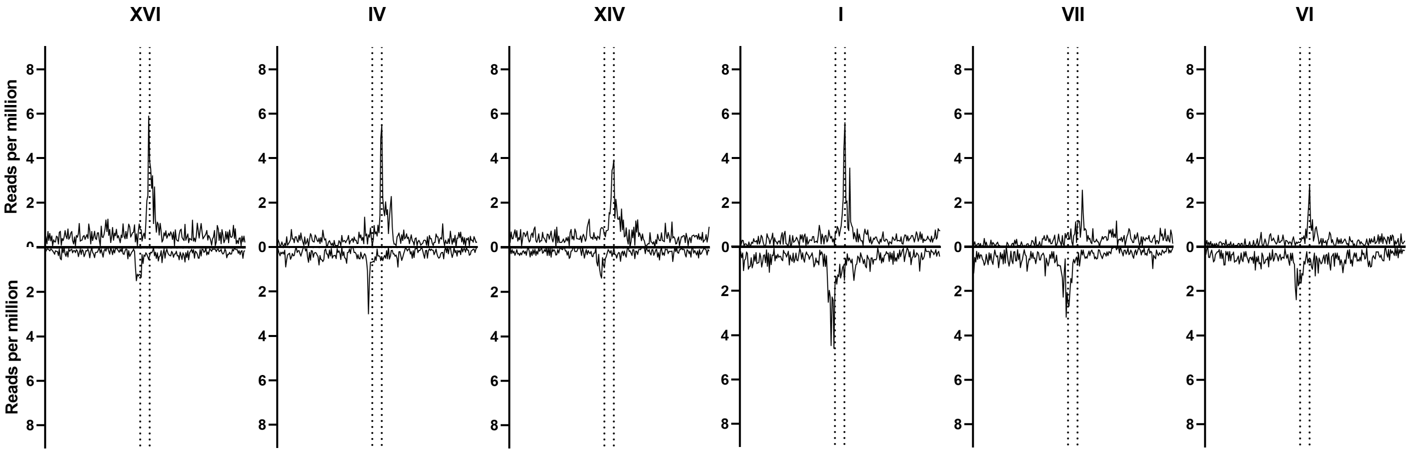

Supplement: S1 File — (PDF) [file pbio.3000886.s008.pdf]
