# Supplementary material for: Genome-wide analysis of DNA replication and DNA double-strand breaks using TrAEL-seq
Source: PLoS Biol. 2021 Mar 24;19(3):e3000886. doi: 10.1371/journal.pbio.3000886 (PMC8021198; doi:10.1371/journal.pbio.3000886)
Supplement: S2 File — (DOC) [file pbio.3000886.s009.doc]

**TrAEL-seq**

Reagents and consumables are detailed at the end of the protocol

*Input material*

Start with agarose plugs containing 1-4x10^7 yeast cells that were ethanol fixed at harvest or 1-2x10^6 mammalian cells[[1]](#footnote-2) (see protocol for Agarose Embedding), you need ½ of a plug per sample.

*Stopping points*

This protocol is very long but can be done in bits, and we have stopped at many points without problems. To our knowledge all the following stopping points are fine:

You can pause indefinitely at any point where plugs are in tris buffer, store at 4°

You can pause indefinitely when DNA is in solution in TE, store at 4°.

You can pause indefinitely when DNA solutions have ethanol added for precipitation, is a pellet in the 70% ethanol, or the pellet is re-suspending in TE. Store at 4°.

You can pause indefinitely when DNA is bound to magnetic beads once beads are in bead wash or TE, store at 4°. Vortex well to re-suspend when you re-start.

Once you start the library preps using the NEBNext Ultra II kit, you must continue until the beads are in the wash solution, but then you can store at 4°.

After eluting the DNA from the magnetic beads with USER, store the libraries in the freezer until ready to amplify. Similarly, amplified libraries before or after purification are stored in the freezer.

*Protocol*

Transfer ½ plug 2ml tube

Equilibrate plug once in 100µl 1x TdT buffer, 30 min at room temperature (RT)

Exchange for 100ul containing:

10ul 10x Terminal transferase buffer (NEB)

4ul 10mM ATP [[2]](#footnote-3),[[3]](#footnote-4)

1ul Terminal transferase (NEB)

84ul water

Incubate 2-3 hrs at 37

Discard the solution and rinse plug with 1ml tris buffer

Then equilibrate plug 1 hr at RT with:

10ul 10x T4 RNA ligase buffer (NEB)

40ul 50% PEG 8000[[4]](#footnote-5)

50ul water

Exchange for 100ul containing:

10ul 10x T4 RNA ligase buffer[[5]](#footnote-6)

40ul 50% PEG 8000

1ul App LIG-seq 3 adaptor (AKA TrAEL adaptor 1)

48ul water

1ul T4 RNA ligase 2 KQ

Mix all components except ligase in a 1.5ml tube, vortex vigorously, then add ligase and pipette repeatedly with a P200 cut tip to mix it really well

Incubate overnight at 25deg [NOT LONGER!!!]

Discard reaction mix, rinse plug 1x 1ml tris buffer, transfer to 15ml tube

During the day wash 4x with 10ml tris buffer at room temperature on rocker

Leave overnight on rocker with 10ml tris buffer

Put plug in normal 1.5ml tube and equilibrate 15min or more with 1ml agarase buffer[[6]](#footnote-7)

Meanwhile, heat a block to 65° and another to 42°C [[7]](#footnote-8)

Remove the supernatant, add 50ul agarase buffer to plug and melt 20 min at 65°

Shift tubes to 42° for 5 min, then added 1ul β-agarase, flick, incubate 1 hour at 42°

Be quick during addition – do not let the tube cool down

Add 25µl 10M NH_4_OAc, 1µl glycoblue and 330µl of ethanol, vortex well

Chill 15 min on ice then spin 15 min top speed 4°

Wash with 70% ethanol, dry 5 min, resuspend in 10µl 0.1xTE, >10 min at RT

For mammalian samples 15 min at 65° is better but make sure it doesn’t go dry

Add master mix containing 29µl water

5µl Isothermal amp buffer (NEB)

3µl 100mM MgSO4

2µl dNTPs (10mM)

1µl Bst 2 warmstart pol (NEB)

Pipette to mix and re-suspend pellet

Heat 30min at 65°

Add 12.5µl 10M NH4OAc, 1µl glycoblue and 160µl of ethanol, vortex well

Chill 15 min on ice then spin 15 min top speed 4°

Wash with 70% ethanol, dry 5 min, resuspend in 130µl 1xTE 15min at 65°

Can proceed directly or store at 4°

Transfer to Covaris tubes

Machine use:

Turn on machine

Turn off and restart!

Turn on computer

Turn on chiller

Fill water tank to 6L

Fill in booking sheet

Protocol (Cris in JHGroup folder)

Duty factor 10

PIP 175

Cycles 200

Temp 11

After use turn everything off, empty water and dry

Wash 8µl Dynabeads MyOne streptavidin C1 beads per sample twice with 0.5ml bead prep buffer (5mM Tris pH8, 0.5mM EDTA, 1M NaCl, 0.05% Tween), 3 min on wheel per wash [you can wash beads for up to 4 samples together]

Resuspend beads in 300µl 2xTN (10 mM Tris pH 8, 2 M NaCl) per sample, split into 300µl aliquots in 1.5ml tubes and add 170µl water to each

Add 130µl sonicated DNA to beads[[8]](#footnote-9)

Incubate on wheel 30 min at RT

Spin tubes up to 1000rpm before putting on magnet to collect liquid

Wash beads 1x 500µl 5mM Tris ph8, 0.5mM EDTA, 1M NaCl, 5 min on wheel

Spin tubes up to 1000rpm before putting on magnet to collect liquid

Wash beads 1x with 500µl 0.1x TE, 5min on wheel

Spin tubes up to 1000rpm before putting on magnet to collect liquid

Carefully remove the wash solution so as not to leave any drops and re-suspend beads immediately in 25 µl 0.1x TE

Store overnight at 4° or carry on directly

Add 3.5μl NEBNext Ultra II end repair buffer

1µl 1ng/ul salmon sperm DNA[[9]](#footnote-10)

1.5μl NEBNext Ultra II end repair enzyme

Pipette to mix and incubate 30 min at 20º (room temperature) then 30 min at 65º

Let cool then spin 10s at 1000rpm

Add 1.25μl new ENDseq adaptor 2 (from annealed 10uM stock)[[10]](#footnote-11)

0.5μl ligation enhancer

15μl NEBNext Ultra II ligation mix

Pipette to mix and incubate 30 min at 20º (room temperature)

Remove reaction mix using magnet, rinse beads 500ul wash buffer (5mM Tris ph8, 0.5mM EDTA, 1M NaCl). REMEMBER your libraries are on the beads!

Wash beads 2x 1ml wash buffer (5mM Tris ph8, 0.5mM EDTA, 1M NaCl) 10 min on wheel each

Spin tubes up to 1000rpm before putting on magnet to collect liquid

Wash beads 1x with 1ml 0.1x TE, 10min on wheel

Spin tubes up to 1000rpm before putting on magnet to collect liquid

Carefully remove the wash solution so as not to leave any drops and re-suspend beads immediately in 11µl 1x TE + 1.5μl USER enzyme

Incubate 15 min at 37º

This elutes the DNA from the beads

Collect beads using magnet, transfer 12µl supernatant to a 1.5ml LoBind tube using an orange LoBind tip

The supernatant contains the library but we do another round of elution to get anything left. This is the first step in which the very low concentration DNA is unprotected, hence the LoBind tubes and tips

Resuspend beads in 10.5µl 1x TE + 1.5μl USER enzyme

Incubate 15 min at 37º

This elutes any remaining DNA from the beads

Collect beads using magnet, add 12µl supernatant to the 12µl from the first elution using an orange LoBind tip

Store samples at -20 if necessary

We then perform a test amplification to work out how many cycles we need to use for the real library.

In PCR tubes, mix 1.25μl library

0.4μl NEBNext any index oligo

0.4μl NEBNext universal oligo

2.95ul water

5μl NEBNext Ultra II PCR mix

98º 30s

98º 10s \

65º 75s / x 18 cycles

65º 5 min

4º hold

Clean with 8μl AMPure beads, elute in 2.5μl 0.1x TE

You can elute up to 2.0μl from these but it is easier just to put the eluate and bead mix on the magnet and pipette 1µl straight onto the Bioanalyzer lane

Run on Bioanalyzer

Use this to calculate the cycle number for the final amplification – note: because of the various dilutions, assume that the final library would be 4x more concentrated than the test library, so reduce the number of cycles by 2 before further adjustments. Try to aim for a final library concentration of 1-2 nM. Adaptor dimers are often seen in these test reactions, be sure to exclude from the quantification.

Final library amplification:

In PCR tubes, mix 21μl library

2μl NEBNext index oligo

2μl NEBNext universal oligo

25μl NEBNext Ultra II PCR mix

98º 30s

98º 10s \

65º 75s / x calculated cycles

65º 5 min

4º hold

Clean with 40μl AMPure beads, elute with 26μl 0.1xTE for 25μl yield

Clean again with 20μl AMPure beads, elute with 11μl 0.1xTE for 10.5μl yield

Run 1µl directly on Bioanalyser and use 0.2µl for KAPA quantification [see separate protocol]

**Reagents**

*Adaptors*

TrAEL-seq adaptor 1:

[Phos]NNNNNNNNAGATCGGAAGAGCGTCGTGTAGGGAAAGAGTGTU GCGCAGGCCATTGGCC [BtndT] GCGCUACACTCTTTCCCTACACGAC GCT

Purchase PAGE purified from Sigma-Genosys or other supplier

5’ App added using the 5’ adenylation kit (see separate protocol for this)

TrAEL-seq adaptor to (aka: ENDseq adaptor 2):

[Phos]GATCGGAAGAGCACACGTCTGAACTCCAGTCUUUUGACTGGAGTTCAGACGTGTGCTCTTCCGATC*T

Purchase PAGE purified from Sigma-Genosys or other supplier

Annealing:

20µl 100uM ENDseq Adaptor 2 in 200ul 1x T4 DNA ligase buffer

Incubate in heating block at 95°C 5 min then remove block from the heat and leave to cool to room temperature

*Reagents and consumables*

Terminal Transferase (NEB M0315S)

10mM ATP (NEB) aliquot on receipt and store at -80°, keep in use aliquot at -30° for no more than 1 month

T4 RNA ligase 2 truncated KQ (NEB M0373S)

T4 RNA ligase buffer (NEB supplied with T4 RNA ligase 2)

50% PEG 8000 (NEB supplied with T4 RNA ligase 2 or home-made as required)

β-agarase (NEB M0392S)

GlycoBlue (Thermo AM9515)

1µl Bst 2 WarmStart DNA polymerase (NEB M0538S)

Isothermal amplification buffer (NEB supplied with Bst pol)

100 mM MgSO_4_ (NEB supplied with Bst pol)

10 mM dNTPs

Ethanol

AFA microTUBEs (Covaris 520045)

Dynabeads MyOne streptavidin C1 beads (Thermo, 65001)

NEBNext Ultra II DNA kit (NEB E7645S), additional Q5 mix may be required which can be purchased separately from NEB

1ng/µl sonicated salmon sperm DNA or other carrier

NEBNext Multiplex Oligos set (e.g. NEB E7335S), additional USER mix may be required which can be purchased separately from NEB

AMPure XP beads (Beckman A63881)

Bioanalyser high sensitivity DNA chip (Agilent 5067-4626)

KAPA qPCR (Roche KK4835) or equivalent library quantification kit

*Solutions*

These solutions are made with milliQ water

Tris buffer 10mM Tris pH8.0

Agarase buffer 10 mM Bis-Tris-HCl, 1 mM EDTA pH 6.5

These solutions are made with certified DNase/RNase free water

10 M NH_4_OAc

1x TE and 0.1x TE Diluted from 100x stock

2xTN 10 mM Tris pH 8, 2 M NaCl

Wash buffer 5 mM Tris pH 8, 0.5 mM EDTA, 1 M NaCl

Bead prep buffer 5mM Tris pH8, 0.5mM EDTA, 1M NaCl, 0.05% Tween

*Equipment*

Magnetic rack for 1.5 ml tubes

Magnetic rack for 0.2 ml tubes (optional)

Covaris E220 sonicator

Other sonicators may be suitable, protocol adjustments will be required

PCR machine

Agilent Bioanalyzer or equivalent gel system

Wheel

Rocker

2 heating blocks

LoBind 1.5ml tubes

LoBind tips for P20

1. Ethanol fixing of mammalian cells yet to be tested [↑](#footnote-ref-2)
2. Stored long term in -80, keep aliquots at -30 for <1 month [↑](#footnote-ref-3)
3. Watch out, we have 1mM, 10mM and 100mM ATP around, get the correct one! [↑](#footnote-ref-4)
4. Use home-made 50% PEG – the kit does not come with enough [↑](#footnote-ref-5)
5. Care! Not T4 DNA ligase buffer! [↑](#footnote-ref-6)
6. Home made, but same as NEB agarase buffer [↑](#footnote-ref-7)
7. Make sure these really reach temperature before use! Give them at least 20 min [↑](#footnote-ref-8)
8. With our current filter tips it is very hard to remove material from Covaris tubes – use a normal yellow tip instead [↑](#footnote-ref-9)
9. This is included to buffer the voracious activity of T4 DNA polymerase in the End repair mix so it doesn’t degrade rare DNA fragments. It may not be necessary. [↑](#footnote-ref-10)
10. Note – do NOT use the ENDseq 2c adaptor – it will not work! [↑](#footnote-ref-11)
