# Supplementary material for: Genome-wide analysis of DNA replication and DNA double-strand breaks using TrAEL-seq
Source: PLoS Biol. 2021 Mar 24;19(3):e3000886. doi: 10.1371/journal.pbio.3000886 (PMC8021198; doi:10.1371/journal.pbio.3000886)
Supplement: S1 Raw Images — Note that not all lanes are presented in the manuscript. (PDF) [file pbio.3000886.s017.pdf]

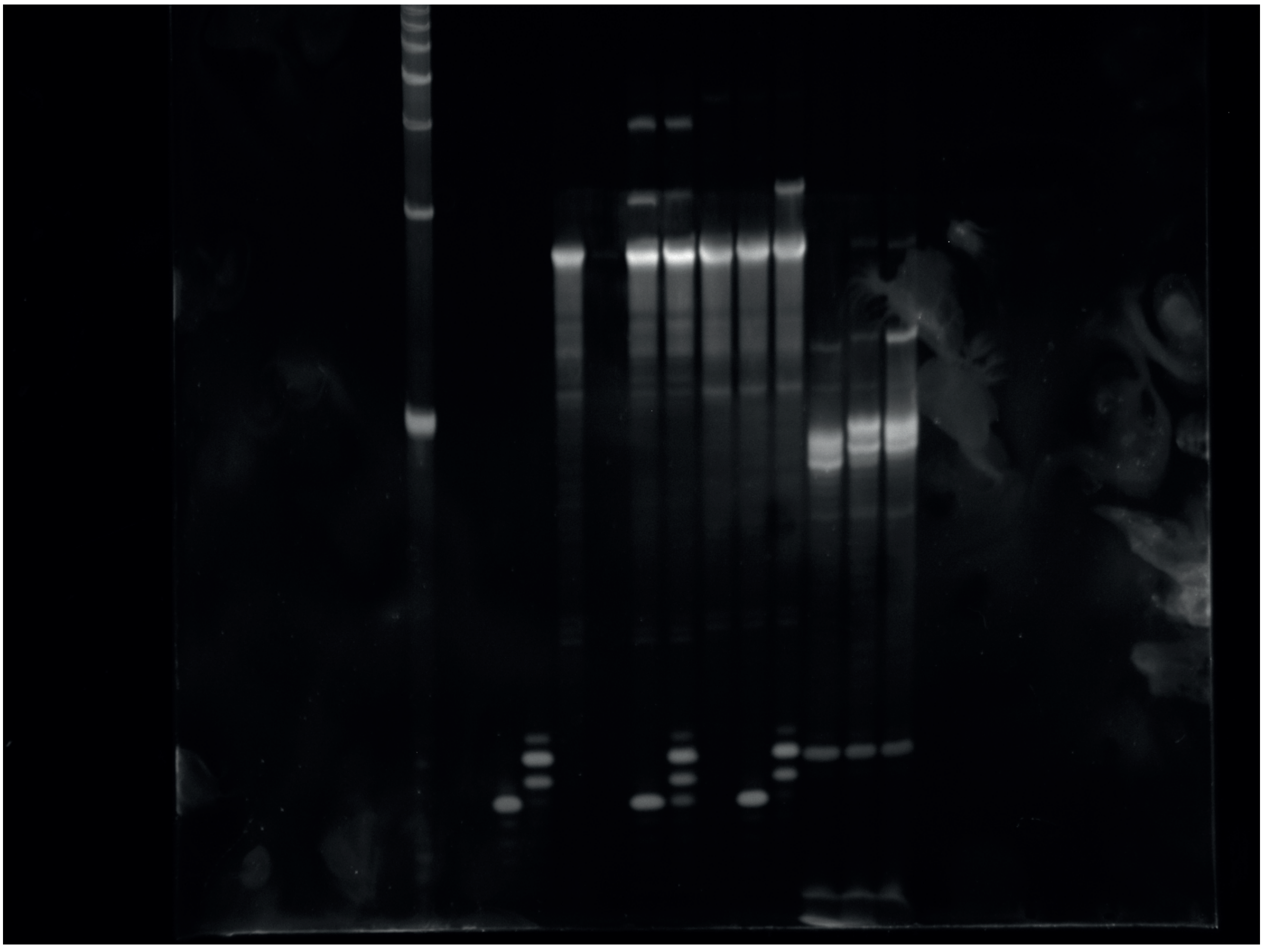

Lanes 1-3 and 7-9 are used in Fig. 1B

Lanes 5,6 are a different a different method we also trialed

Lanes 10-12 are tests of the USER cleavage of the adaptor
