# Supplementary material for: Genome-wide analysis of DNA replication and DNA double-strand breaks using TrAEL-seq
Source: PLoS Biol. 2021 Mar 24;19(3):e3000886. doi: 10.1371/journal.pbio.3000886 (PMC8021198; doi:10.1371/journal.pbio.3000886)
Supplement: Attachment — Submitted filename: Response to TrAEL reviewers.docx [file pbio.3000886.s018.docx]

**Response to TrAEL-seq manuscript reviews**

We would like to thank all the reviewers for their helpful comments. The experimental and textual changes that you requested have substantially improved the quality of the manuscript. We have improved clarity in many places, analysed G1 arrested yeast to demonstrate replication dependence of TrAEL-seq profiles, and added other experiments and analysis as requested.

We have also updated some datasets that we felt should be improved. In Fig. 2D/E and 3A/C/D (original numbering) we had previously used the yeast wild-type and mutants from Fig. 2B to show TrAEL-seq reproducibility. Now we have substantially more libraries, we have chosen to show 3 biological replicates of wild type that were generated at different times by different workers using different reagent batches, and include both the best and worst libraries we have made. These are obviously less tight than the previous data, but all show the effects we describe and better represent the spread of TrAEL-seq data. We have also generated more replicates of the undifferentiated hESC data so we now show 2 biological replicates in 2C and 3H, each formed of two technical replicates, and we have removed the retinoic acid treated data as this was not adding any useful information.

**Reviewer #1:**

The authors introduce a method for the genome-wide detection of DNA 3' ends that they call TrAEL-seq. The initial experiments clearly demonstrate the sensitivity of the approach to detect free 3' ends generated by restriction enzyme cuts. The authors then go on to assess the pattern of meiotic double strand breaks, sites of replication fork stalling and the pattern of DNA replication. Intriguingly, and somewhat frustratingly, the authors don't have an explanation for why TrAEL-seq can be used to determine the direction of replication forks. Overall, TrAEL-seq looks to be a useful addition to the wide range of methods available to assess DNA ends and DNA replication.

I have a number of comments that I hope might help the authors improve their manuscript:

1. This manuscript is primarily a methods paper and therefore I think it is important that a number of additional controls are considered:

- for the second half of the manuscript the authors use TrAEL-seq to assess DNA replication. However, I didn't spot an experiment that shows that the signal the authors are detecting is DNA replication dependent. A simple TrAEL-seq on arrested yeast cells (e.g. in alpha-factor) would provide clear evidence that the output reported is replication dependent. There are multiple places in the manuscript where the authors describe the signal and state that it is DNA replication dependent, however without a non-replicating control I don't see how they can support these statements. Furthermore, there is likely to be some level of background (there is to any genomics method) and a non-replicating control should allow the authors to assess this. This would be particularly important when the authors compare mutants or growth conditions (glucose vs. other carbon sources), since the fraction of S phase cells is very likely to be different and therefore background signal could be influencing the results.

This is an excellent experiment, but one that proved difficult to perform. The problem is one of quantitative comparison between libraries that have no fixed point (the same problem often encountered comparing between ChIP-seq datasets for example). To achieve this, we implemented a modified TrAEL-seq protocol in which two samples (G1 arrested cells and cells released into S) were ligated to two different TrAEL-seq adaptors carrying two different barcodes. The samples were then mixed prior to the Bst polymerase step and processed as a single library, then unmixed after read mapping and deduplication. For each experiment two libraries were generated with the barcodes swapped to ensure that barcodes did not impact quantitation. This approach allows quantitative comparisons between libraries. The results, in the new Fig. 3E, show strong replication profiles from the cells released into S, but few reads were obtained from the G1 cells.

This shows that TrAEL-seq signals are replication dependent and that the background in non-replicating cells is really remarkably low. In fact, even the background in the G1-arrested cells is phased (see image below) and we therefore suspect that most of the reads in G1 samples actually stem from the small contamination of replicating cells that are inevitably present in alpha-factor arrested populations of *BAR1* wild type.

This browser screenshot shows read polarity plots for chromosome V. The G1 profile (top) is identical to the S phase profile (bottom), but very noisy as it is derived from very few reads.

- that authors use restriction with SfiI to introduce the method. These were well thought through and well executed experiments. This is an enzyme that leaves a 3' overhang. At various points through the manuscript the authors mention that TrAEL-seq has differing sensitivities to various 3' ends (e.g. figure 2A coloured dots). It would be useful for the authors to properly assess this by performing a TrAEL-seq experiment with a cocktail of various enzymes that leave different produces (nicking, 5' overhand, 3' overhand, blunt ends).

This was a good experiment with rather unexpected results. We digested a plug of wt DNA with NotI, PmeI and SfiI which produce 5’ overhang, blunt and 3’ overhang respectively, and processed half the plug each into END-seq and TrAEL-seq libraries. In contrast to previous reports of TdT activity on different types of end, the selectivity was not very strong – END-seq was more sensitive to NotI sites as expected whilst they both performed similarly on SfiI sites. Unexpectedly, TrAEL-seq performed better than END-seq for blunt *Pme*I ends. We have replaced Fig. 1C with this new experiment, and re-performed the matching SfiI end analysis in Fig. 1D using this new data so that the experiments match (the results in Fig. 1D did not noticeably change). We have modified statements about selectivity towards overhanging 3’ ends in the light of this experiment.

Activity on nicks is much more difficult to assess, as we do not have GLOE-seq implemented in the lab and END-seq does not detect nicks meaning that we lack a positive control. We used qPCR to test for ligation of TrAEL adaptor to a defined nick introduced by Nb.BsrDI; we could detect this but only just separated from the background (which comes from replicating DNA in the sample); the caveat being that it is very hard to confirm Nb.BsrDI nicking efficiency by qPCR. Therefore our analysis suggests that sensitivity to nicks is low but proper quantification will require further investigation.

However, sensitivity to nicks or otherwise inaccessible 3’ ends cannot explain the replication profiles that we observe as if that were the case then GLOE-seq, which is excellent for nick detection, would give stronger replication signals than TrAEL-seq. In fact, the converse is true for replication and other signals – we have found no signals in wild-type GLOE-seq libraries that are stronger than in TrAEL-seq and that could therefore be assigned to detection of nicks. We have added this point to the discussion of why TrAEL-seq detects processive replication forks.

- the authors undertake a comprehensive analysis of the bases detected at the 3' end of the SfiI digested material. I would like to see the authors undertake a comparable analysis to see if there is any preference for the base at or close by to the detected 3' end when TrAEL-seq was applied to various genomic DNA substrates.

We have added this data as Fig. S1C, based on the sequences detected at the 3’ ends of the fragments generated by *Sfi*I cleavage. We see no bias based on the identity of the 3’ terminal or adjacent nucleotide.

- how much does the TrAEL-seq read density across the genome vary? In Fig. 3B (and similar figures) the authors present the strand bias, but not the total number of reads mapping to each region. This would be a useful analysis, particularly when considering what might be the source of the 3' ends detected by TrAEL-seq.

We have added read density data to complement Fig. 3B as Fig. 3D. We note that read density is generally uniform across chromosomes, with pronounced dips at each ARS (further evidence that this signal is primarily replication derived), and a peak at the centromere (as seen in Fig. 2D).

In figure 4B the density on the reverse strand is shown and appears to have some periodicity; I'd encourage the authors to look further as to the nature and potential source of this periodicity.

We agree there is some periodicity – this is particularly pronounced in the average plots around TSSs that we have added as Fig. 4D. The peaks are roughly nucleosome spaced, however we don’t want to over-interpret these as we haven’t found any informative mutants that change the signal, and don’t have a good explanation of why TrAEL-seq signals should phase with nucleosomes. We have noted this however in the Results section describing these plots. It is also worth noting that this signal is nowhere near as strong as the equivalent phasing seen in GLOE-seq data (Fig. 4B of Sriramachandran et al), but it is not really clear why GLOE-seq data from wild-type cells should phase with nucleosomes either.

2. The authors don't discuss the difference between wild-type and rad52-delta cells at centromeres (rad52-delta gives reduced peaks) and tRNA genes (rad52-delta seems identical to wild-type). Why is the signal at centromeres reduced in the rad52 mutant?

We have found that this resulted from a problem with the normalisation – there is no real difference. We have generated new plots comparing wild type to rad52 that are now in Fig. S2E (the main panel plots now show 3 biological replicates of wild-type cells which we think are more useful).

3. The authors speculate as to why TrAEL-seq has the ability to report on replication fork direction, but (as they clearly state) they don't have a confirmed molecular mechanism. As such, I think they should be more careful in their terminology in some places, particularly in referring to the y-axis of plots such as Fig 3B, D, F & G as "Replication Fork Direction". They are not measuring replication fork direction (and don't have a explanation for why what they are measuring correlates with replication fork direction) and therefore this shouldn't be used to label these axis. The authors should find an alternative description, such as "strand bias" for the manuscript text and figure labels.

Yes, this is reasonable. We have changed RFD to Read Polarity as that is how it is described in the text.

4. Are the authors confident that RNase T1 (used to treat DNA in plugs) cannot digest adjacent to ribose bases incorporated into genomic DNA? If it can this could offer an alternative explanation for the observed strand bias that allows the authors to infer replication fork direction. However, it would also require the authors to reassess the rnh201-null/rnh202-null experiments, since in this case they would not be reporting what the authors think they are.

We think this is very unlikely as RNase T1 is specific to single-stranded substrates. Furthermore, even if RNase T1 accessed individual DNA strands during transient melting, this enzyme is highly specific to guanine ribonucleotides whereas we detect no bias towards 3’ guanine at cleavage sites (Fig. S1C). Finally, RNase T1 cleavage leaves a 3’ phosphate that would prevent tailing by TdT or ligation by T4 RNA ligase as both enzymes require a 3’ OH substrate.

5. What is the difference between the dnl4/rad51 double mutant and rad52-delta data?

Very little in practice, but we used these mutants for slightly different reasons. *RAD52* deletion should block all HR after resection, giving the maximum chance of detecting normally transient HR intermediates; given that DSBs at the RFB occur in S or G2 when HR is the primary repair pathway, this mutant would be predicted to capture the widest variety of events. In reality we capture no events above wild-type, hence our conclusion that signals detected at the RFB are not DSBs repaired by HR. In contrast, breaks caused by transcription of the *GAL* genes could occur in G1 when NHEJ is a more important repair pathway. To capture all breaks in this situation we created a double mutant that blocks both HR and NHEJ; ideally we would have used a *dnl4 rad52* mutant but we were not able to make this mutant at the time for unknown reasons. A *dnl4 rad51* mutant should have an equivalent effect in these experiments.

6. In the results the authors suggest that "the replication fork is stalled by chromatin or proteins bound at the promoter" when the GAL genes are induced. The authors should test this hypothesis by looking in their other datasets at the promoters of constitutively highly expressed genes.

This is a good point. We have added average plots around the TSS for the top 25% expressed genes in YPD (based on published NET-seq data), separating genes into those head-on or co-directional with the replication fork (Fig. 4D). We see a sharp peak just before the TSS for head-on genes that is absent from co-directional genes, and also from head-on genes in the bottom 25% expression category. As with the GAL genes, the peak is not striking, but is reproducible in high quality libraries so we think this is real.

7. The authors have a highly plausible explanation for how 3' ends might report replication fork direction: "The mechanism responsible must frequently rearrange replication forks to make the leading strand 3' end accessible to TdT while the lagging strand 3' end remains largely inaccessible. We suggest that transient fork reversal would have this effect..." First, the use of the word "must" here seems too strong given how many unknowns remain (for example, see suggested control experiments above). Second, I suspect that this transient fork reversal is more likely to take place in the plug after Proteinase K treatment rather than in cells. The DNA is treated with Proteinase K at 50 °C which should allow for transient melting of the free 3' end (on the leading strand) and thus interconversion between the species drawn out in figure 5. Both the elevated temperature and the removal of proteins would seem to make this interconversion more likely than in cells.

We take the reviewer’s point, and agree that it is very difficult to know if these events occur *in vivo* as heating and de-proteinisation are currently necessary for TrAEL-seq labelling. We have therefore reworded the first paragraph of the section entitled “*A proposed mechanism for replication fork detection by TrAEL-seq*“ to reflect the potential for the fork reversal events to occur during processing.

Finally, the authors use green and red dots to distinguish certain features (fig 2A & 5), plus possibly green and orange violins (fig 4E). I'm red/green colour-blind and the colours used are indistinguishable to me. I don't know what the PLoS family of journals policy is on the use of colours accessible to all, but I'd encourage the authors to change this. More information and advise is available here: <https://thenode.biologists.com/data-visualization-with-flying-colors/research/>

We thank the reviewer for pointing this out. We think we have changed all the problematic colour schemes, but please let us know if we have missed any!

Minor comments:

- introduction: "This can be a problem as end resection forms extended tracts of 3' extended single stranded DNA..." I think this could be more clearly phrased to avoid using the word "extended" twice.

Changed extended to long in one instance.

- introduction: "However, these methods do not have the resolution to detect individual origins unless markedly different in timing, and a range of other more specialised approaches have been applied to study replication initiation..." This is not correct. In budding yeast (the organism used for most of the presented work) copy number approaches are more than sufficient to detect individual replication origins. Furthermore, copy number approaches are very much more straightforward than the approach presented in this paper.

We have clarified that we were talking about mammalian cells in this sentence.

- results: "This was ligated with ~10% efficiency to pre-adenylated TrAEL-seq adaptor 1 using truncated T4 RNA ligase 2 KQ, a ligase that is specific for 5' adenylated adaptors (Fig. 1B)". I did not understand what the authors were doing here; why was the adaptor pre-adenylated and why are they interested in a 5' adenylated adaptor when TrAEL-seq uses a 3' adenylated substrate?

We have removed the reference to oligo adenylation here as we see it is confusing. 5’ adenylation of adaptors is a widely used technique to improve ligation specificity, and does not really need to be mentioned here as it is detailed in the methods section. It is completely unrelated to the 3’ adenylation performed by TdT that is critical for TrAEL-seq, it is an unfortunate coincidence that the terminology ends up being identical.

- results: "...consistent with previous studies that reported both co-directional and head-on tRNA transcription can stall replication forks, at least in the absence of replicative helicases (Fig. 2E arrows) [65-67]." The authors have missed a pre-genomics study from Carol Newlon's group:

Deshpande, A. M., & Newlon, C. S. (1996). DNA replication fork pause sites dependent on transcription. Science (New York, NY), 272(5264), 1030-1033. <http://doi.org/10.1126/science.272.5264.1030>

This paper uses DNA 2D gels to detect replication fork pausing at a tRNA genes in wild-type cells.

This was cited in the previous sentence (as citation 64), but we have added a citation here as well.

- results: "There should never be less 3' ends on the lagging strand..." less should be fewer.

Changed.

- results: "ARS211" should be in italics.

Changed

- discussion "existing methods profile either fork direction or origin timing" - I think that recently developed single molecule nanopore methods, such as D-NAscent, can profile both fork direction and origin timing.

We thank the reviewer for pointing this out. We have added the sentence following sentence to this paragraph, and slightly rephrased the following point to emphasise that the ability to work without synchronisation or label incorporation improves flexibility.

“The recently described D-Nascent method can determine fork direction and origin timing, but only after cell synchronisation and label incorporation.”

- fig 1A: it would be useful for the authors to label at least a couple of the free ends with 5' and 3'.

Done.

- fig 1B: which lanes contain substrate? What's the difference between lanes 4 & 5? Or 0 and 2?

We have added labelling for the substrate and adaptor to the top of the gel as well as the side to improve clarity.

- fig 1E: I think that a further 'zoom in' on one of the peaks would be a valuable addition.

We have added a further zoom in for the major peak showing the data at nucleotide resolution.

- fig 2D: why have the authors presented a meta-analysis of the signal across the various classes of centromeres? It would be useful for the authors to show each individual centromere in the supplement.

We think that splitting the centromeres into classes usefully summarises this data. However we agree that some readers would find the individual plots more useful so we have added these in S1 File.

- fig 3G: why have the authors reversed the y-axis for the human GLOE-seq data, but not the yeast?

This is a good point. We have now flipped the polarity of both as it makes the comparison easier.

- fig 4B: it would be valuable for the authors to show the forward strand reads in addition to the reverse strand reads.

We have added this data. There are almost no reads on the F strand as this whole region is replicated left->right.

- fig 4C: I'm not sure I fully understand how the data has been normalised for these two plots. What is responsible for the asymmetry? There seem to be many more locations where the read count is lower (e.g. below -50) than higher (e.g. above +50).

Only a minimal normalisation for total library read count has been applied. The asymmetry results from more sites showing a large reduction in read count on transition to galactose, but these are still a tiny minority compared with the vast majority of sites (in the red region of the heat map) that are unchanged. Of course, on average, this majority population must be fractionally higher on galactose to balance the smaller number of sites showing a large decrease, but this effect is not visible when spread over so many sites (there are >1 million data points on each of these graphs).

- fig S1A: a scale bar should be added.

In light of comments from the other reviewers, we have removed this panel; these assays were overly technical for this paper.

- fig S1B: it is not clear what the x-axis scale is. Is this the difference in cycle number compared to the control?

Again, this has been removed as overly technical for this paper.

- fig S2B: why is there such a large difference in peak height between the two conditions?

We need to investigate this further – it is not clear whether the replication fork progression changes on treatment and that is really beyond the scope of this work. For now, we have removed the retinoic acid condition entirely from the paper (this condition was an added complication that did not add to our conclusions) and have concentrated on ensuring the reproducibility of the unstimulated hESC data for which we have added further technical and biological replicates.

**Reviewer #2:**

In this manuscript, Kara and colleagues describe a novel NGS-based assay to map DNA double-strand breaks called Transferase-Activated End Ligation sequencing (TrAEL-seq). This method adds to the long list of assays (BLESS, BLISS, Break-seq, END-seq, i-BLESS, GLOE-seq…) that have been recently developed to map single- and double-strand DNA breaks and have revolutionized the fields of DNA repair and genome stability. The authors convincingly show that this method can efficiently capture single stranded DNA 3' ends genome-wide in yeast and in human cells. From this respect, this method differs from BLESS and related assays (BLISS, END-seq, i-BLESS…) in that it maps the real cleavage site and does not require the degradation of the 3' overhang, which can be several kb long. Yet, TrAEL-seq is not just another method to map DSBs. Indeed, the authors show that TrAEL-seq provides also a very sensitive and accurate method to map replication fork direction (RFD) in unsynchronized populations of cells. The quality of RFD maps provided for yeast and human ES cells is very impressive. It seems to match the performance of more labor-intensive assays such as OK-seq without requiring the use of ligase mutants. Moreover, the authors show that TrAEL-seq can be used to map programmed pause sites at the rDNA of yeast and human cells and at tRNA genes with an unprecedented resolution. They propose that the ability of TrAEL-seq to map paused and moving forks is due to frequent fork reversal, exposing a 3' overhang on the leading strand that can be extended by TdT to capture it.

Beyond the validation of the assay, the manuscript provides convincing evidence that transcription does not induce replication fork pausing in budding yeast, which remains a controversial issue (see specific issues below). It also shows that changes in carbon sources in the growth medium alters the position of replication termination sites, presumably by altering the timing of origin firing. It also shows that the replication fork barrier is polar in hESCs, as it is the case in yeast and unlike in human cancer cell lines.

Overall, the manuscript is very well written and the data are clear and convincing. The experimental pipeline is well described and the detailed protocol provided in supplementary materials will be very useful to those interested in implementing TrAEL-seq in their laboratories. Importantly, the authors also provide advices on how to combine TrAEL-seq with other methods and stress the fact that many of the existing DSB mapping assays are complementary and should be combined to embrace the complexity of DNA damage and repair processes. In conclusion, I have no doubt that this novel assay will be quickly adopted by a wide community and especially to those working on DNA replication and genomic instability. However, the following issues need to be addressed before publication.

Specific issues:

1. Page 1 and 2, the authors review the existing NGS-based assays to map DNA breaks but omitted Break-seq, an assay developed by the Feng lab (Hoffman et al, 2015, Genome Research 25, 402). Is there any reason for that? If not, it would be fair to mention this method together with the others.

We did not include Break-seq originally as it was not clear that this method can distinguish breaks from single stranded DNA regions. However, the recent Cell Reports paper from Chakraborty *at al.* published after submission of our manuscript provides substantial further validation, showing nucleotide resolution mapping of breaks. We have therefore added both Break-seq papers to the relevant paragraph of the introduction.

2. Modifications of BLESS (page 2) have not only improved ligation efficiency, but also increased the signal-noise ratio. As discussed in ref 15, the main limitation of the original BLESS assay was a very high noise caused by artifacts associated with formaldehyde fixation.

We have clarified the text to note specifically an improvement to signal-to-noise for i-BLESS. However we did not want to delve too deeply into the differences between the individual methods except where they are directly relevant to TrAEL-seq.

3. One of the most striking features of TrAEL-seq is its sensitivity for replication forks. The author's explanation that limited fork reversal occurs very frequently even at unchallenged forks is plausible. Another likely possibility that is not discussed by the authors is that branch migration or partial unwinding of nascent DNA occurs after DNA extraction. Would it be possible to experimentally address this possibility?

We cannot rule this out at the moment, and have now added discussion of this possibility in the first paragraph of the Discussion section titled “*A proposed mechanism for replication fork detection by TrAEL-seq”*. We have not found a way to convincingly test this experimentally as yet – we have tried to perform TdT tailing on permeabilised cells prior to heating and deproteinisation but this was ineffective.

4. Figure 4B shows that highly expressed PolII genes do not induce replication fork pausing, unlike tRNA and rRNA genes. This observation argues against earlier studies in budding yeast (e.g. Azvolinsky et al., 2009, Mol. Cell 34, 722) but is consistent with a more recent study showing that spontaneous replication stress in yeast is not caused by replication-transcription conflicts (Forey et al., 2020, Mol Cell 78, 396). This issue should be discussed in the manuscript.

Yes. We have added the following sentence to the paragraph in the results section in which Fig. 4 is described: “Our evidence for minimal replisome pausing even at the most highly expressed genes contrasts with previous estimates based on DNA polymerase or γH2A occupancy [76, 77], but is in keeping with more recent studies that have not observed defects in fork progression or activation of Mec1 when replication forks encounter highly transcribed genes [67, 78].”

5. Figure 4B also shows that breaks occur at the promoter of GAL7 and GAL10 genes upon galactose induction. Do these breaks depend on DNA replication or are they also detected in G1 cells?

This is a good question that we have addressed using our new quantitative G1/S phase data, as described in the response to reviewer one’s first point. We have added new data (Fig. 4D) showing that a peak upstream of the TSS in highly expressed genes orientated head-on to the replication fork is not exclusive to the GAL genes and is seen in average plots of all highly expressed genes. Repeating such plots with the G1 and S-phase data shows that the signal is uniformly lower across the TSS region in G1 cells with no pronounced peak (Fig. S4E). Therefore, this peak is specific to S-phase, and most likely depends on replication. The dependence on replication direction also strongly supports this assertion.

6. Although the conclusion that ~2% of the yeast genome shows altered replication direction between glucose and non-glucose media is valid, the way Fig. 4E was built is unclear. This figure aims at comparing RFD in cells grown on different carbon sources "by filtering windows for those with a difference in RFD>0.4 between the two sets". However, several strains grown on glucose (fob1, rad52, rnh201, rnh202, clb5) were not tested on raffinose and reciprocally for dln4 rad51, so it is not clear which two sets were compared to select windows with a RFD>0.4. For instance, what is clb5 grown on glucose compared to? If it is to wild type cells on raffinose, it is misleading to assume that the difference observed is due to the carbon source. Since samples are not labeled in the figure, it gives the impression that authors are comparing apples and pears. The authors should rather restrict the main figure to wild type cells grown on glucose, raffinose and raffinose + galactose and

move the figure with the whole set of data to supplementary material after clearly explaining how samples were compared to each other. Focusing the main figure on wild type cells would also stress the fact that the main determinant of this RFD difference is the absence of glucose and not the induction of GAL genes.

We agree that the comparisons we made were not well validated. We have retained the original description that we find the sites of difference by pooling cells grown in different media independent of mutation as this was the logic of the analysis. However, we have expanded our dataset such that Fig. 4E now purely focuses on wild-type and clb5 cells, and we have included data for clb5 grown on raffinose (which looks very similar to clb5 on GLU in this analysis), such that for both wt and clb5 we have both GLU and non-GLU datasets. The analysis was re-performed using our newer datasets, but the result is essentially the same.

**Reviewer #3:**

General comments:

1. The overall clarity of the paper could be improved by making it clear in Figure 1A that TrAEL-seq reads are the opposite polarity of the actual inferred 3' end. Throughout the paper, I was occasionally confused by this, until noticing in figure caption 1A that "TrAEL-seq reads map antisense to the cleaved strand, reading the complementary sequence starting from the first nucleotide before the cleavage site.". This description itself is confusing and would be clarified with a better and more complete figure in Figure 1A (and elsewhere.

This is a good suggestion – we have added a step to Fig. 1A showing the actual reads with a statement that they are the reverse complement of the original reads. We also state in the text of the reorganised results:

**“**TrAEL-seq libraries are sequenced in single end mode using a primer that anneals to TrAEL-seq adaptor 1, and the sequence read is the **reverse complement of the original DNA 3’ end**.”

An exact depiction of the sequence reads and relationship to the 3’ end is now given in Fig. S1B.

2. Abstract. Please revise extensively to make the text appropriate for a general non-specialist audience (e.g. PLoS Biology). The opening the abstract requires some general information about DNA replication (what it is, what it is for, what the problems/questions are) that sets the scene. As written it jumps in too fast to a technical aspect of DNA replication fork stalling. Indeed, to a non-specialist it is not even clear the text is talking about *DNA* replication.

Yes, on re-reading we agree with this point. We have added introductory sentences and removed some technical details from the abstract.

3. In general, please take time to split the text up more into smaller paragraphs with more headings, and make sure each experimental section is appropriately introduced. As currently written, it will confuse a lot of the PLoS Biology readership. Currently, too many things are left unstated. Please be explicit

We have re-worked much of the results section and tried to improve the introductions and explanatory sections. We hope this is now much clearer.

---

Page 8. Major comments:

1. The authors contend that they are able to map nascent replication forks. If this is correct, the authors should test by preparing libraries in alpha-factor arrest (or G2/M arrest). A more complex experimental test (that may not work) would be a release into S-phase and demonstrate both that signals arise and also move in the expected direction of replication.

This is an excellent experiment, but one that proved difficult to perform. The problem is one of quantitative comparison between libraries that have no fixed point (the same problem often encountered comparing between ChIP-seq datasets for example). To achieve this, we implemented a modified TrAEL-seq protocol in which two samples (G1 arrested cells and cells released into S) were ligated to two different TrAEL-seq adaptors carrying two different barcodes. The samples were then mixed prior to the Bst polymerase step and processed as a single library, then unmixed after read mapping and deduplication. The results, in the new Fig. 3E, show strong replication profiles from the cells released into S, but very few reads were obtained from the G1 cells. Barcode swapped datasets were included to ensure that the different barcodes do not have an effect.

It is clear from the data that release from G1 arrest was not very synchronous and so we do not see the signals moving away from the origins – rather the profile simply appears at the G1->S time-point we chose based on bud sizes. This is something that we are keen to pursue further, but will require both improvement of the adaptor barcoding to allow multiple timepoints and a more rigorous arrest-release protocol to tighten the cell synchronisation. This was not possible in the timescale of these revisions and will need to feature in a follow-on manuscript.

2. The authors suggest that they are detecting transient instances of fork reversal. Whilst possible, it strikes me as highly unlikely that such events would be so frequent and so prevalent as to generate the data in Fig 3. I would like to suggest an alternative explanation: That they are detecting instances of fork collision with Top1 on the leading strand ahead of the fork. Please refer to: <https://www.ncbi.nlm.nih.gov/pmc/articles/PMC85758/>

The above paper demonstrates how only Top1 CCs on the leading strand template generate DSBs. Indeed this model could explain the strand disparity observed by TrAEL-seq. (Note that the Top1-CC would prevent detection of signal with the opposing polarity, because the Top1-CC would prevent ligation.).

This was an interesting idea that we have tested, but we do not see any effect on TrAEL-seq signal in a *top1*Δ mutant. We have added this data to Fig. 3G and S3D.

3. The authors do not appear to have normalised their data for relative sequencing depth; thus any comparisons between libraries/methods are circumspect and misleading as presented. Please: A) Tabulate the read depth and mapping/filtering etc of all libraries. B) Present the consistency/correlation of repeat TrAEL-seq datasets to justify pooling. (I assume there are biological duplicates and that they were pooled in some way?)

Yes – we accept that the data was not as clearly presented as it should have been. To address this we have:

1. Normalised all the presented data to reads/million mapped to help comparisons
2. Added direct comparisons between technical replicates for dmc1, wt, and hESC samples in Figs. S1-3.
3. Added data showing reproducibility of replication profiles and replication fork stalling data in multiple biological replicates for both yeast and hESCs
4. Included S2 Table describing all the samples used and the relationships (technical or biological) between them.

---

Other specific comments:

Page 2. "DNA breaks cannot therefore be mapped post-resection by BLESS-type methods, which is problematic as DSB repair is often easiest to inhibit post-resection (such as in classic rad51Δ or rad52Δ mutants in yeast)." This statement needs revising: This is absolutely not true (as written). DSBs can be (and have been!) mapped, but lack nucleotide precision of the original site.

Agreed – this sentence did not convey our point. Have changed to

“The original location of DNA breaks as opposed to the end-point of end resection cannot therefore be mapped by BLESS-type methods,”

Page 2. "Profiles yielded by BLESS-type methods can rarely be considered in isolation as replication has a dramatic influence on the distribution of DNA strand breaks in a cell;" Is there a reference to back up this very strong ("dramatic influence") statement? Has BLESS (or other) been performed in strains arrested versus going through replication? If so, please reference, and or amend the statement to make it clear that it is the authors' view that the distribution will be altered based on knowledge of fork-stalling/blocks etc.

Yes – both qDSB-seq and i-BLESS were tested for cells entering replication in high concentrations of hydroxyurea. These experiments yielded very strong signals around replication origins. We have added the references to these two papers to this sentence.

Page 2. "Methods have also been developed to detect replication fork directionality through isolation and sequencing of Okazaki fragments (OK-seq) [32, 33]" Since a fairly exhaustive discussion of general methods are being presented, I suggest also including Pu-Seq (<https://www.ncbi.nlm.nih.gov/pmc/articles/PMC4789492>).

Yes – this was inadvertently omitted, so we have added the following sentence: “As an alternative, PU-seq uses a relatively simple DNA library preparation to identify leading and lagging strands based on ribonucleotide incorporation, but does require very specific DNA polymerase mutants with reduced ribonucleotide discrimination [36].”

Page 2. Please hyphenate "single-stranded DNA".

Done.

Page 3. First two paragraphs. The structure of the paper here is very confusing. It opens with a description/overview of the method (and mentions the use of T4 RNA ligase), but then in paragraph 2 'implementation", the text reads as if the RNA ligase has not yet been mentioned. This is very confusing. Please revise the text so that the order is logical and suitable for a broad readership. In general, many of the paragraphs/sentences could be greatly improved with simple leaders such as: " In order to do X, we did Y...".

We agree that this was confusing. In response to this and later comments we have extensively re-ordered and streamlined these first sections to improve readability

Page 3. Is the efficiency of tailing and ligating to a (very) short 18 nt oligo a suitable way to measure the efficiency of tailing and ligating to rare ssDNA ends within large genomic DNA fragments? What is the estimated relative molarity of ends in the test vs real reaction?

Absolutely – *in vitro* assays like this are critical to ensure that complex labelling reactions work as designed. However, to allow visualisation these inevitably use higher concentrations of substrate than are present on real genomic DNA - we calculate ~1000x more ends in this amount of oligo versus an *Sfi*I-digested plug. We would expect that the lower levels of substrate in genomic DNA would drive up the tailing and ligation efficiency, but this is very hard to measure.

MINOR: Fig 1 legend. Please don't refer to a ssDNA as 18 bp (base pairs) in length when there is no base pairing. It is 18 nucleotides (nt) in length.

Absolutely. Fixed.

Page 3. Please define ∆Ct in the main text. To maximise accessibility, please avoid all unnecessary jargon and abbreviations.

Page 3 "DNA was digested prior to ligation with the restriction enzyme SfiI, then ΔCt was calculated for qPCR reactions that detect TrAEL adaptor 1 ligated to an SfiI cleavage site in the ribosomal DNA (rDNA) (Fig. S1A)."

Confusing sentence structure, please revise to:

"DNA was digested with the restriction enzyme SfiI prior to ligation TrAEL adaptor 1, then ΔCt was calculated for qPCR reactions that detect TrAEL adaptor 1 ligated to an SfiI cleavage site in the ribosomal DNA (rDNA) (Fig. S1A)."

Page 3. "Data from two experiments was compared with two libraries previously generated using an END-seq protocol..."

This is very unclear. What is meant by previously generated? Are these published data from somewhere? Or new by the lab? If this is the first time these data have been presented, why does the text state "Previously generated"? What point is being made by such a statement?

Minor. Figure 1B, there is no description of what the short vertical bars and dots indicate. (I presume means and raw datapoints.)

Page 3. The final paragraph is very technical and hard to follow. The figure legend for S1C does not help at all.

In response to all these points and our own re-reading of the manuscript, we agree with the reviewer that this part of the text was too technical for a PLOS Biol paper and really wasn’t adding anything. We have therefore removed this text and associated figures. We have streamlined the first three results sections for better readability.

In addition, Presentation of a single bioanalyser trace in Fig S1D is unhelpful if the point is to demonstrate primer removal: the input lanes need to also be shown. (Note, I'm really not sure that this panel and the text is needed at all...but in its current form it is of very limited value.)

We don’t fully agree with this point. When implementing a new method, we find that it is extremely useful to have example data for quality control steps such as this one, as well as the final sequencing data. We have added an arrow to show where adaptor dimer peaks appear when present. We cannot show the input lane as it wasn’t run – the output from the PCR would have far too strong primer dimer peaks for this to be useful anyway. However, we note that this panel has changed as we have swapped the original *Sfi*I data for improved restriction digestion data based on the experiment requested by reviewer #1.

Was the genomic DNA digested to completion? Or was it partial? Where is the raw data supporting this?

*Sfi*I, *Not*I and *Pme*I are effective enzymes for digestion in agarose plugs (<https://www.neb.com/tools-and-resources/usage-guidelines/digestion-of-agarose-embedded-dna-info-for-specific-enzymes>), and we use substantially more enzyme per unit DNA than in these tests. qPCR across the 18S cleavage site that we have used for QC across development of this method showed a reduction of 8 cycles in *Sfi*I-digested material, corresponding to a digestion efficiency of >99%.

Page 4. "Comparing TrAEL-seq data for SfiI digested genomic DNA to an END-seq library generated from equivalently digested material shows high concordance (Fig. 1C)." To aid reader comprehension, the opening sentence of this section should first indicate that TrAEL-seq libraries were sequenced, then briefly describe how the data were processed (i.e. mapping, genome, tools used, and any filtering etc) before comparing with END-seq data. This is especially confusing (as currently written) since all prior data presentation concerned qPCR at specific test loci.

We understand this point, but we think that adding this detailed bioinformatic data to the main text would detract from the clarity of the text. Instead, we have added a detailed schematic of the bioinformatic pipeline as Fig. S1B. We have extensively re-written these early sections of the manuscript anyway and removed all the qPCR validation, so we hope that the reviewer agrees that the presentation is now much clearer.

Page 4. What does "unambiguously" refer to here? How would the data look if the 70 SfiI sites had been detected "ambiguously"?

We have removed this word as it was not helpful.

Page 4. Without further details about how mapping, trimming and/or filtering were performed (summarised clearly in the main text), interpretation of the nucleotide accuracy is circumspect. For example, How many rA bases are assumed to have been added? Are all T bases trimmed prior to (or after) mapping?

1-3 rA bases are assumed. No, not all T bases are trimmed as this would be problematic for breaks that occur in genome-encoded poly(A) (see next answer).

Page 4. "We suggest that this overall mapping accuracy of >99% within ±1 nucleotide would be sufficient for almost all applications." This estimation is only relevant for DSBs generated by SfiI. DSBs from other sources may form at loci with longer runs of genome-encoded adenosines, where the algorithm may not perform as well. (Actually, what is the algorithm that has been used? Why is it not explained in the main text?) This sentence is therefore misleading, as it implies that the algorithm accuracy (">99% within ±1 nucleotide") is true for "almost all applications". Please address this by estimating accuracy at sites with longer runs of genomic-encoded adenosines or by revising the interpretation/presentation of this accuracy estimation (and by explaining how this algorithm works).

Apologies - we did not explain this clearly. We had already considered the problem of over-trimming in genome-encoded A tracts, and therefore limited the trimming to 3 nucleotides maximum (the average tail length). We have added a sentence directly addressing this point, and data in Figure S1D to show that the 10 *Sfi*I sites that have more than one consecutive A at the 3’ end are not over-trimmed.

Page 4. "A major strength of TrAEL-seq should be the ability to map DSB sites after resection," AND "This shows that TrAEL-seq accurately maps endogenous resected DSBs.". These sentences could be clarified to indicate that TrAEL-seq maps the original site of DSB formation, rather than the endpoint of resection.

We have clarified these sentences.

Page 4. "Meiotic DSBs formed by Spo11 are processed by Sae2 amongst other factors prior to resection, after which strand invasion into a sister chromatid is mediated by Dmc1 [40, 41]." In meiosis, homologous recombination occurs between homologous chromosomes, rather than sister chromatids. Please revise.

That was a silly error – we thank the reviewer for pointing this out! Corrected.

Page 4. "TrAEL-seq for resected DSBs in dmc1Δ cells 7 hours after induction of meiosis revealed a DSB pattern very similar to that observed for unresected DSBs in an sae2Δ mutant mapped by S1-seq (a BLESS variant specific for meiotic recombination) (Fig. 1E)." Please cite the paper from which the sae2D S1-seq data originate.

At time of writing this data is unpublished – the Keeney lab generously shared this with us (as we note in the Acknowledgements). However, the data is now available from the GEO database, and the accession number is noted in the Figure legend.

Page 4 and Figure 1F. "Across all hotspots for Spo11 cleavage, quantitation of DSB usage frequency by TrAEL-seq correlated well with S1-seq (R2=0.86) (Fig. 1F, left), and to a similar extent with Spo11- associated oligonucleotide sequencing (R2=0.84) (Fig. 1F, right)". These correlations whilst useful are rather crude comparisons. Since an advertised benefit of the method is the nucleotide accuracy, a more detailed comparison showing how the nucleotide accuracy of S1-seq and TrAEL-seq compares within a zoomed-in strong hotspot region is expected. Indeed, since much is being made of the single-nucleotide accuracy, the authors should compare their dmc1 TrAEL-seq to meiotic CC-seq data (Gittens et al 2019 <https://www.nature.com/articles/s41467-019-12802-5>), which, unlike Spo11-oligo seq, has no mapping ambiguity (no tailing is involved).

We have added a nucleotide resolution panel to Fig. 1E.

We have attempted the comparative analysis requested but the results are uninformative. The problem being that Spo11 sites do not occur at tightly defined nucleotides but rather in clusters over 50-500 bp. Spo11 cleaves at many sites with very complex patterns within these clusters (see illustrative figure below). TrAEL-seq reads do not line up very well at this resolution with CC-seq, S1-seq or Spo11 oligo seq, but the disparity is no worse than between the other methods and this disparity is far greater than can be explained by the minimal tailing involved in spo11-oligo seq. We suspect that these patterns vary with the exact SK1 variant and culture conditions used; this will only be resolved by a full side-by-side comparison of these methods, which is beyond the scope of this work.

This figure shows a browser snapshot of read densities plotted at nucleotide resolution across the region lying 5’ of *ARE1* (the strongest hotspot visible in Figure 1E). Datasets (top to bottom): TrAEL-seq *dmc1*Δ, CC-seq *sae2*Δ, S1-seq *sae2*Δ and Spo11 oligo-seq. Genomic position on chromosome III is visible at the bottom. The differences in read position resulting from the different methodologies (a matter of a few nt’s each way) have been compensated where possible. The position on Chr. III is shown under the bottom track.

Additionally, an important point about sensitivity is missing here: How many of the 3901 hotspots were detected by TrAEL-seq? How does this compare to the number detected by S1-seq (and/or CC-seq datasets)?

We have added this analysis to the text: we defined a cut-off for detecting the known Spo11 sites based on signal exceeding the mean + two SDs of the signal from random equivalent sized regions in the rest of the single copy genome. By this metric, TrAEL-seq detects 3542 out of 3907 sites (89%), which is higher than S1-seq (2556) or CC-seq (3223), but this comparison is deceptive as the *sae2*Δ mutant used for S1-seq and CC-seq suppresses many Spo11 sites (PMID: **18076285). Spo11 oligo sequencing is far superior to any of these methods, detecting 3784 of the sites, but is far more demanding of time and material. Considering the effects of the *sae2*Δ mutation, we suspect that TrAEL-seq is not superior to CC-seq for detecting Spo11 cleavages, though both methods seem to outperform S1-seq.**

Specifically, the Y-axis of Fig 1F is about 5-fold lower in TrAEL-seq. Assuming all reads are expressed as hits per million mapped reads (or equivalent? *Please add this information to all figures*), then this strongly suggests that the signal to noise of TrAEL-seq is significantly lower than S1-seq, which itself is lower than Spo11-oligo seq.

**This just stems from our plotting raw read counts on these graphs which we agree was not very helpful. We have re-plotted these as reads per million, as we have throughout the paper.**

Page 5. "Two RFB sites are readily visible in wild type TrAEL-seq data as peaks of antisense reads relative to the direction of replication" AND "At centromeres replicated from one direction only, we observed an accumulation of reads antisense to the direction of replication just before the centromere, while forks in termination zones that can be replicated in either direction displayed both peaks (Fig. 2D)."

These sentences are unclear. The use of "sense" and "antisense" is typically in relation to transcription, yet replication is mentioned instead. This section could be clarified by referring to the orientation of the 3' DNA end itself, relative to the leading/lagging strand. E.g. add: "…, corresponding to 3' DNA ends on the leading strand".

**We agree this was unclear and have rephrased without the use of the words sense/antisense to be absolutely specific.**

More specifically, do peaks on the R-strand indicate nascent (genomic) 3' ends on the F or R strands? Please revise text to make sure this information is absolutely clear throughout the manuscript.

Always on the F strand – as now clarified on Fig. 1A and in other places, the reads are always on the opposite strand to the original DNA end.

Page 5. "although TrAEL-seq data displays higher signal-to-noise ratios than GLOE-seq data with peaks that correspond more closely to known sites than qDSB-seq peaks (Fig. 2B) [13, 36]."

Where is the evidence for greater signal to noise in TrAEL-seq vs GLOE-seq? Perhaps the second replicate of GLOE-seq is worse...but what is the cause of this? Were TrAEL-seq replicates highly correlated? Where are these data demonstrating that fact (both genome-wide, and specifically at this locus?). Reproducibility of TrAEL-seq is a very important point that must be presented and commented upon.

Apologies for this – we did not make our point very clearly. By signal-to-noise we were referring to the frequency of additional peaks that are not in the known RFB sites and are not conserved between replicates. Even in the first GLOE-seq replicate, such peaks are more prominent on both strands. We have re-phrased this to be clearer.

We agree that we had not shown sufficient data on reproducibility. To directly address this, we have added comparisons between wildtype yeast technical and biological replicates at the RFB to show reproducibility (Fig. S2A, S2B and S3B). We also note that we have altered the datasets presented in figures 2D, 2E, 3A, 3C and 3F to show 3 biological replicates of wild-type, giving a good insight into the real variability of the method. Similarly, we have added replicates for the hESC samples and included comparisons between biological and technical replicates in Figs. 2C, S2D, 3H, S3F and S3G

Page 5. "It should be noted that the TrAEL-seq and the GLOE-seq datasets used for this analysis derived from asynchronous cells whereas the cells for qDSB-seq had been tightly synchronised in S phase, underlining the high sensitivity of TrAEL-seq for stalled replication forks."

Please revise. Just because TrAEL-seq detects a signal in the asynchronous cells says nothing in of itself of the sensitivity compared to other assays (which is the impression teh text is trying to make). Specifically, the synchronized qDSB-seq signal is at least 20-fold stronger. If the authors want to compare the sensitivity, they should synchronise their cells to make the data comparable.

This issue partially arises from the use of raw read counts which we have now changed (see previous comments). However we have removed the statement about S-phase synchronisation as it is not critical to the argument.

However...I am now additionally concerned that the authors have not normalised their data for total sequencing depth. This is essential if any comparisons are to be made between the various samples and sequencing techniques: All data need to be presented as reads per million mapped.

Agreed and done. This does not change any of the conclusions but does allow better comparisons.

A table stating the read depths and mapping statistics of all individual libraries used here is also *essential*.

We have included this information in the new S2 Table.

Page 5. "Importantly, no difference was observed between the rad52Δ signal and the wild type, showing that these double stranded ends are not normally processed by the homologous recombination machinery (Fig. 2B compare wild type and rad52Δ panels)." As stated above, this comparison is only valid if read depths have been normalised between samples. Please clarify and revise the interpretation if not.

As noted in the figure legend, the read counts between these libraries is very similar so normalisation has no effect on this result – the *rad52*Δ signal remains slightly lower than the wild type. However, the data is now normalised as suggested and the result is unchanged.

In general the logic of this section is very poorly introduced and explored. The authors make far too many implicit jumps in data interpretation without fist introducing the concepts that they are testing (i.e that if this signal is a reversed fork, one would expect Rad52 dependence). This needs radical revision to be acceptable text for a broad journal like PLoS Biology: in its current form, non-specialist (and even specialist) readers will be lost. The fact that the peak is similar (assuming still true after normalisation) in the presence and absence of Rad52, needs more careful interpretation and clearer description. The jump to Holliday junction processing makes no sense without greater, slow, detail explaining the logic.

We have carefully re-written this section to improve clarity.

Page 5. "To determine the applicability of TrAEL-seq to mammalian cells, we generated TrAEL-seq libraries from 0.5 million human embryonic stem cells (hESC) that were either undifferentiated or subjected to retinoic acid-induced differentiation." What is the purpose of the retinoic acid-induced differentiation here? Do the authors expect to see a difference at the rDNA?

We were curious as to whether a simple treatment that initiates differentiation causes a difference in the TrAEL-seq profile. We saw little evidence of this, but included this data as in the initial submission we had little data from human cells (our initial submission timing was forced by the publication of the GLOE-seq paper, while at the same time our experiments were very limited by the COVID lockdown). We have replaced the retinoic acid data in this revised manuscript with further technical and biological replicates of untreated hESCs.

Furthermore, the data here is incredibly noisy, and far from convincing. How many other genomic sites shows such similar peaks? What methods do the authors have to demonstrate that these signals are real and not noise? What was the total read depth of these human samples?

We have improved this data and now show profiles from two biological replicates, each being an average of two biological replicates. Indeed, the data is noisy compared to yeast, but it should be noted the RFBs in human cells are far less prominent than in yeast. The peaks that we observe are in very good accord with published 2D gel data from the Kobayashi lab (PMID **25776556). We have sequenced these to ~50 million reads per library, but actually most reads are lost in deduplication so sequencing deeper would not improve the data greatly.**

How is mapping accuracy validated within the R repeats? Can the spike be an artefact stemming from copy number differences between the sample and reference? Or due to mis-mapping of reads to the repeat array? All these details are completely glossed over, leaving this reviewer far from convinced by the data presented.

Yes – this is a concern. The three repeats are very similar, though contain a few SNPs different that should support mapping. Nonetheless, we would indeed expect some level of mis-mapping between the repeats, although this should tend to equally distribute reads across the three repeats rather than focus them in a single repeat as we observe.

To probe this further, we compared a hESC TrAEL-seq library to a total DNA library prepared from the same plug of cells (so exactly matching). In the browser screenshot below, the top row is the TrAEL-seq data for the rDNA, reverse reads only orientated as in Fig. 2C, with a single prominent peak at the first R repeat.

A general mis-mapping to the repeat array should equivalently manifest in total DNA – the middle row shows the total DNA profile that we have run through exactly the same bioinformatic pipeline, and no peak is visible.

Conversely, the UMI-deduplication can create artifacts in multi-mapping regions. The bottom row shows the same data which has not been UMI deduplicated, and has instead been deduplicated purely based on read start, ie: if 2 or more reads map to the same site, only one is kept. This is a very crude deduplication that is not capable of artifactually increasing signal in multi mapping regions. Again, the peak is unaffected.

We think that these tests are sufficient to show that this signal is real and not a mapping artefact.

Page 5 and Fig 2DE. The three colours are incredibly hard to distinguish. Why are they not labelled in the plot itself? (why only in the figure legend?). Is the reader meant to interpret anything from the differences (or not) between these mutants?

We agree that these were hard to read, but have decided instead to re-plot these graphs using 3 biological replicates of wild-type. This better conveys the main point (that the signals are very reproducible), and removes the need to analyse each individual line. We have then added clearly labelled figures comparing wild-type to rad52 in S2E Figure.

I also have concerns that only smoothed data (I assume that is what the wiggly lines are?) is plotted rather than nucleotide-resolution peaks of the raw signals.

The problem is that the nucleotide resolution data is very noisy. The replication forks do not pause at nucleotide-precise sites, so there is nothing gained by showing the nucleotide resolution data. However, we take the reviewer’s point that the smoothing is deceptive, so we have re-drawn these plots using un-smoothed data in non-overlapping 10 nt bins.

Fig 2E. The signals at the tRNA are intriguing, but lead to a query: Is there any possibility that nascent RNA can act as a 3' end for TrAEL-seq? From Fig 1A, I assume a 3' RNA end would efficiently ligate to the first adapter, and also be reverse-transcribed by the Bst2.0 polymerase. From the methods it appears that adapter 2 is 5' phosphorylated, thus would be able to ligate to the new DNA 3' end generated by Bst2.0 (even though it would be a putative a DNA/RNA hybrid molecule). This product would then be a substrate for PCR because a single DNA strand would have been created with adapter 1 on the left and adapter 2 on the right. I would like the authors to respond to this and explain how they can exclude this as a molecule that they may be inadvertently detecting using TrAEL-seq. I recognise that the authors use RNAseT1 in their DNA preparations, but I do not know if this enzyme cleaves the DNA/RNA hybrids present at sites of nascent transcription that I am suggesting may a source of 3' RNA ends.

We understand the concern here as RNase T1 will not degrade RNA in RNA/DNA hybrids, and mechanistically this suggestion is plausible. However, the reads forming the peak at the 5’ end of tRNAs are of the wrong polarity to arise through attachment of adaptor 1 directly to the tRNA or a fragment thereof. If adaptor 1 was ligated to the 3’ end of an RNA, the sequencing read (which is primed from adaptor 1) would be the reverse complement of that RNA; as fig. 2E is orientated, this would mean that reads from direct ligation of Adaptor 1 to the tRNA would be on the reverse strand whereas actually they are on the forward strand.

Separate form these comments above, where is the evidence that the peak in the tRNA gene is replication dependent (and thus a site of replication stalling?). The peak in the tRNA is similar in both local replication orientations. Could it instead be (if not a labelled RNA species), a site of increased DNA template breakage due to the high levels of transcription going on here?

We completely agree and state this in the manuscript:

“…we also observed a major peak covering the first ~15bp of the tRNA gene, which was not affected by replication direction and appears to mark a transcription-associated break on the template strand…”

Page 6. The section: "A replication signal of the same polarity was noted...yet up to 90% of TrAEL-seq reads emanate from the leading strand." Is incredibly hard to follow in relation to the preceding text figures, as the authors now refer to the position of the 3' end (I think?) (rather than the antisense TrAEL-seq read). I would suggest that throughout the paper/figures, the inferred position of the 3' end is exclusively referred to, for clarity. This is how the data were presented in the GLOE-seq paper, which will also aid comparison by the reader.

We have attempted in all cases to ensure that we specify whether we are talking about the reads themselves or the position of the DNA 3’ end that is indicated by that read, and have gone through the manuscript to make sure we are absolutely specific.

However, we think it is critically important in a methods paper to represent the real data, in this case the read polarity, without distorting it even where that may help comparison with a particular previous work. We were very confused by data presented in the GLOE-seq paper until we noticed the statement in the analysis section that the strand of all the reads had been switched. One case in which this is critical is in answer to the reviewer’s earlier question about whether tRNA signals derive from ligation of adaptor to the RNA; answering this requires data presentation to be completely unambiguous about the polarity of reads obtained. We hope that our data presentation is now unambiguous, but are happy to add further clarifications where needed.

Please clearly summarise/conclude this section: Is TrAEL-seq detecting nascent 3' ends of the leading strand? If so, please state this clearly! If this is the case, why are nascent 3' ends on the lagging strand not also detected? (there should be many more of them too).

We have added the following statement to the summary paragraph for this section:

“The 3’ ends detected by TrAEL-seq correspond to the leading rather than the lagging strand, despite the fact that many more 3’ ends occur on the lagging strand, and we suggest that these 3’ ends become exposed by replication fork reversal either *in vivo* or during sample processing.”

Yes. The fact that we detect only leading strand ends despite there being many more on the lagging strand was a major piece of evidence leading to the fork reversal model in Fig. 5. As we note in the discussion where we derive this model:

“…The mechanism responsible must frequently rearrange replication forks to make the leading strand 3’ end accessible to TdT while the lagging strand 3’ end remains largely inaccessible.”…”

Please also define RFD in the legend.

We have now removed references to RFD in response to reviewer 1’s comments. We instead show the same data as read polarity, which more clearly shows the direction of the reads.

In general, it seems inappropriate to refer to the plot in Fig 3B as an RFD plot (really it is just a strand ratio), since at this point it is not clear what signal TrAEL-seq is detecting.

This is not unreasonable – we have changed the term to “read polarity” on graphs and in the text, and added arrows to the Y-axes showing that positive and negative refer to more reverse and forward reads respectively.

Page 6. "suggesting that double stranded ends are also formed during normal replication". Please consider that ssDNA/dsDNA junctions are likely to be mechanically fragile (even in agarose), and may preferentially break during processing generating a structure labelled by END-seq.

This is a good point, and the reviewer is right that we cannot exclude this given the weakness of the signal. We have added the caveat “…although it is possible that this very weak signal may arise from rare replication fork cleavage events occurring in vivo or during sample processing”.

Page 6. "…although with the opposite polarity as expected (Fig. 3F and S3D) [36]." Incorrect figure references. I think the author is referring to Fig. 3G and S3E.

Fixed.

Page 7. "The TrAEL-seq profile of clb5Δ was very similar to wildtype across most of the genome, but certain origins were clearly absent or strongly repressed, resulting in extended tracts of DNA synthesis from adjacent origins (Fig. 4A, green arrows). This is as predicted for clb5Δ mutants and confirms that TrAEL-seq is indeed sensitive to changes in replication profile." The clb5 data are convincing. Could the authors strengthen the manuscript further by adding a supplementary figure showing a global analysis of the differences between wild-type and clb5∆ RFD plots, relative to the differences between two samples not expected to have differing origin usage? E.g. two scatterplot analyses.

Yes, thank you for suggesting this. We have added scatter plots of wt versus clb5 and wt versus rnh201 in Fig. S4A. The clb5 is dramatically different from the wild-type whereas the rnh201 is highly correlated.

Page 7. "stalling but not in the expected location." Please clarify what the expected location was...the reader will be completely lost at this point without a more careful, and slower, presentation of ideas, observations, and interpretations.

To try and improve clarity, we have replaced this sentence and explained our expectations as follows:

“Collisions would seem most likely where the replisome passes through the transcribed region of highly expressed genes oriented head-on to the direction of replication (such as *GAL10* or *GAL7*), so we predicted that any consequent replication fork stalling would occur at the 3’ end of the gene or within the open reading frame. However, TrAEL-seq read densities across the *GAL* gene cluster provided evidence for transcription-associated replication fork stalling but not in the expected location. Peaks of reverse reads formed at the 5' end of the *GAL10* gene, and also of the *GAL7* gene although the latter was less prominent, which suggests that the replication fork is stalled by chromatin or proteins bound at the promoter after passing through the body of the gene”

Fig 4B. This figure is not very convincing. Why are only R-strand reads presented? PLease add an X- scale. What smoothing was applied to the data? (Was it a rectangle or a Hann window, please specify) What was the justification for such a smoothing window? Were the data normalised for relative read depth between the Raffinose and R+Galactose libraries? If the data in 4C are 100 bp bins

We only showed the R strand as no differences were observed on the F strand, but the reviewer is right we should show this and so we have added the F strand data. The smoothing arises only as we have quantified the data in 100bp sliding windows that are spaced at 10bp – no further processing was applied. This is described in the figure legend. Yes these data were normalised for relative depth between the libraries using a minimal correction for maximum library size, this was required for the analysis in 4C, which uses exactly the same quantification data.

Page 8. "Together, these data show that replication profiling by TrAEL-seq is sufficiently sensitive to reveal subtle differences in fork direction and processivity." Where is the evidence to indicate that what is detected by TrAEL-seq is "subtle"? By what benchmark are these subtle effects?

Removed “subtle”.

Page 8, discussion: "Here we have demonstrated that TrAEL-seq maps resected DSBs," Here and elsewhere in the text, I think it is important to clarify the text to make it clear that the TrAEL-seq technique maps the 3' end of resected DSBs. i.e. the presumed DSB end.

Done

Figure 1. "Note that TrAEL-seq reads map antisense to the cleaved strand, reading the complementary sequence starting from the first nucleotide before the cleavage site." This is unclear because the diagram depicts a DSB, where both strands are cleaved. Which is the "cleaved strand"? Could the figure be labelled more clearly to indicate the first sequenced base.

Apologies – this makes no sense! Corrected to “…from the first nucleotide of the DNA 3’ end”. We have added an indication of the sequencing reads to Fig. 1A, while Fig S1B now shows the exact relationship between the reads and the original DNA ends.
