# Supplementary material for: Genome-wide analysis of DNA replication and DNA double-strand breaks using TrAEL-seq
Source: PLoS Biol. 2021 Mar 24;19(3):e3000886. doi: 10.1371/journal.pbio.3000886 (PMC8021198; doi:10.1371/journal.pbio.3000886)
Supplement: Attachment — Submitted filename: Response to TrAEL reviewers 2.docx [file pbio.3000886.s019.docx]

I wonder if the title might be slightly easier to parse if you use "using" instead of "by." Or flip it round to give "TrAEL-seq is a method for genome-wide analysis of DNA replication and DNA double-strand breaks"...

Thanks, yes – we agree and have changed to “using”.

As you address these items, please take this last chance to review your reference list to ensure that it is complete and correct. If you have cited papers that have been retracted, please include the rationale for doing so in the manuscript text, or remove these references and replace them with relevant current references. Any changes to the reference list should be mentioned in the cover letter that accompanies your revised manuscript.

We have proofed the paper again and made some very minor corrections in the manuscript and some of the figures (Figs. 2, 4, S2, S3, S4). We have also added a citation (number 45) – the Keeney lab had shared unpublished S1-seq data with us (used in Fig. 1E/F), this has now been published and we have therefore cited the work. Finally, the first author now has an ORCID account that we have added to the address information.

*Published Peer Review History*

Please note that you may have the opportunity to make the peer review history publicly available. The record will include editor decision letters (with reviews) and your responses to reviewer comments. If eligible, we will contact you to opt in or out. Please see here for more details:

<https://blogs.plos.org/plos/2019/05/plos-journals-now-open-for-published-peer-review/>

Opt in please

*Early Version*

Please note that an uncorrected proof of your manuscript will be published online ahead of the final version, unless you opted out when submitting your manuscript. If, for any reason, you do not want an earlier version of your manuscript published online, uncheck the box. Should you, your institution's press office or the journal office choose to press release your paper, you will automatically be opted out of early publication. We ask that you notify us as soon as possible if you or your institution is planning to press release the article.

Opt in please

DATA POLICY:

Regardless of the method selected, please ensure that you provide the individual numerical values that underlie the summary data displayed in the following figure panels as they are essential for readers to assess your analysis and to reproduce it: Figs 1CDEF, 2BCDE, 3ABCDEFGH, 4ABCDEF, S1CDEF, S2ABCDEF, S3ABCDEFGH, S4ABCDE.

We have uploaded excel files (S1-S7_Data). Sequencing data is publicly available through GEO.

BLOT AND GEL REPORTING REQUIREMENTS:

The gel image is submitted in S1_raw_images.

REVIEWERS' COMMENTS:

Reviewer #1:

[identifies himself as Conrad Nieduszynski]

I would like to commend the authors on the excellent work they have undertaken to address reviewer comments. The manuscript is much improved and I believe ready for publication.

Thank you Conrad – we really appreciate the time you have invested in this.

Minor comment: "although TrAEL-seq data contains less additional peaks in this region than" should be "fewer" rather than "less".

Changed.

In the revised manuscript the authors make the observation of phasing between TrAEL-seq signal and nucleosomes. This is precisely what I was anticipating seeing. Replication fork velocity is stochastic (just compare the length of tracks seen in multiple double pulse labelled combing/fibre studies), but the source of this variability is unknown. Multiple lines of evidence suggest this is sequence independent. Therefore, a possible explanation is that DNA replication frequently 'pauses', if only very briefly, as the fork pushes through nucleosomes (and as new Okazaki fragments are primed). I suggest that this would provide an explanation for the authors observation on phasing. Following this up is far beyond the scope of this manuscript, but in the future should be possible with various mutants that alter the position of nucleosomes and/or the ability of the fork to replicate through nucleosomes.

Reviewer #3:

The authors have done an excellent job answering the extensive comments and suggestions that were provided during the review process. The rebuttal comments are measured and well explained. The updated text and figures have improved clarity. I thank the authors for the time spent providing additional analysis, comparison to other methodological techniques, and for providing a detailed breakdown of the datasets presented in the study (in particular to include statistics on mapping and de-duplication). My main concern with data presentation was clarity on which strand was being referred to, and normalisation of all data to reads per million mapped. Both changes have been incorporated. The authors additionally provide G1-arrested, and even a very exciting attempt at the G1 > S release. Both sets demonstrate clearly the S-phase dependence of the mapped signals.

I have one minor comment/suggestion that I think will aid a non-specialist reader. Would it be possible to make the diagrams of the rDNA in Fig S3B and Fig 2B more consistent with one another? Specifically, how do the three RFBs referred to in Fig 3B (and the two strong TRAEL-seq peaks) relate to the single RFB (and dispersed TRAEL-seq peak profiles) in Fig S3B? Why do the data look so different?

Fig. S3B shows a whole rDNA repeat (9.1kb) whereas Fig. 2B zooms in on the RFB region. To make these figures more consistent we have included an inset panel on Fig. S3B showing where the region shown in Fig. 2B is located.

Also, it is not stated, but is the orientation of the rDNA repeat drawn consistent with the orientation in the standard S. cerevisiae reference sequence? This is probably obvious to the authors, but since there are no chromosomal coordinates indicated in Fig 3B, it is not clear, and thus perhaps this information can be added to the legend.

Yes it is, but we have added chromosomal coordinates to Fig. 2 to make this clear.

Overall, this is an interesting and potentially very useful analysis method that expands the tools available to researchers in a new and sensitive way. The authors should be commended on its development, and on their manuscript.

Thank you – your detailed comments were incredibly helpful in making this manuscript ready for publication.
